# Unveiling Structure‐Activity Relationship With Structurally Defined and Stabilized Cu‐Based Molecular Hybrid Electrocatalysts in CO_2_ Electroreduction

**DOI:** 10.1002/exp2.70231

**Published:** 2026-10-13

**Authors:** Jian‐Hao Wu, Zhi‐Mei Luo, Rong‐Jie Guo, Jia‐Wei Wang, Bingbao Mei, Fu‐Jun Niu, Lie‐Jin Guo, Gangfeng Ouyang

**Affiliations:** ^1^ School of Chemical Engineering and Technology and Southern Marine Science and Engineering Guangdong Laboratory (Zhuhai) Sun Yat‐sen University Zhuhai China; ^2^ Instrumental Analysis & Research Center (Zhuhai) Sun Yat‐sen University Zhuhai China; ^3^ Shanghai Synchrotron Radiation Facility Shanghai Advanced Research Institute Chinese Academy of Sciences Shanghai China; ^4^ School of Advanced Energy Sun Yat‐sen University Shenzhen China

**Keywords:** C‐C coupling mechanism, Cu‐based electrocatalysts, electrochemical CO_2_ reduction, metal‐ligand cooperativity, structure‐activity relationship

## Abstract

Deciphering the structure‐activity relationship on Cu‐based electrocatalysts is critical to rationally optimize their performances in CO_2_ to multicarbon (C_2+_) conversion, which nonetheless remains highly challenging due to their ill‐defined and electrochemically unstable structures. Herein, this study explores Cu‐based molecular hybrids with defined structures to establish structure‐activity relationships in CO_2_‐C_2+_ electrocatalysis, where a Ni(II) phthalocyanine anchored on Cu_2_O nanocubes via 2‐(3‐thienyl)ethoxy groups outperforms its Co(II) analog and unmodified prototype in C_2+_ synthesis (78.3% selectivity, 330 mA^.^cm^−2^), rivaling top catalysts. Structural analysis revealed the functional 2‐(3‐thienyl)ethoxy anchors pose superior Cu_2_O affinity to retain the Cu(I) structure and the grafted metal phthalocyanines during catalysis. Such stabilized and well‐defined catalyst structures enable the structure‐activity relationship unveiled by in situ spectroscopies and theoretical calculations. The results show that the synergy between the molecular metal centers and surface Cu atoms fine‐tunes the energy barriers for both CO transfer and *OCCO hydrogenation, which are minimized by the optimal Cu‐S‐Ni coordination endowing abundant Cu(I)Cu(0)‐paired active sites with an electron‐rich Ni center. This work demonstrates the essential role of metal‐ligand cooperativity in C‐C coupling and successfully bridges mechanistic insights with catalyst design for efficient CO_2_‐C_2+_ conversion.

## Graphical abstract

Schematic presentation of our concept in utilizing the more structurally defined and stabilized Cu‐based molecular hybrids to bridge the gap between the spectroscopic mechanistic findings and the structure‐activity relationship in Cu‐catalyzed ECO_2_R to C_2+_ products.

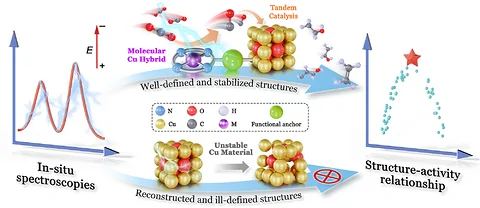

## 1 Introduction

The electrochemical CO_2_ reduction (ECO_2_R) offers a promising pathway for enabling the production of valuable chemicals from CO_2_ using electricity generated from renewable sources [1, 2, 3, 4]. Among the various ECO_2_R products, the deeply reduced multicarbon (C_2+_) products, such as ethylene, ethanol, and propanol, have attracted significant attention due to their high energy densities [5]. However, selective ECO_2_R for specific C_2+_ products remains a key challenge, fundamentally owing to the substantial energy barriers in coupling C‐C bonds and the competitions with other pathways [6]. Cu‐based materials have been regarded as the most effective catalysts in this term by their appropriate binding affinity of *CO for facile C‐C bonding [5]. To understand the C‐C coupling mechanism, a handful of mechanistic studies have revealed the key involvement of CO dimerization processes and subsequent reduction/protonation [7, 8, 9, 10, 11, 12, 13, 14, 15]. More intriguingly, state‐of‐the‐art in situ vibrational spectroscopies combined with density functional theory (DFT) calculations have emerged to be effective for online monitoring the potential‐dependent CO dimerization and detecting the key *C_2_ intermediates (e.g., *OCCO, *OCCOH, *OCHCH_2_, etc.; asterisk denotes the adsorbed state) toward final products, therefore proposing possible mechanisms [16, 17, 18, 19, 20, 21]. However, although progress has been made to address the challenges in deciphering the complex mechanism of Cu‐catalyzed C‐C bond formation [12, 15, 22, 23, 24, 25, 26, 27], little research is able to utilize these mechanistic insights to elucidate the structure‐activity relationship of the Cu‐based electrocatalysts for their further theory‐guided optimizations, which ought to be the fundamental purpose of the above mechanistic studies.

Such gaps from the mechanistic knowledge to the structure‐activity relationship among the Cu‐based inorganic catalysts [20, 25, 28, 29, 30, 31, 32] or Cu‐centered coordination compounds [33, 34] can be partially attributed to their disordered and/or dynamic structures under electrochemical conditions [20, 25, 30, 31, 32, 35]: it is challenging to correlate the measured catalytic activity and/or detected intermediates with their ill‐defined configurations or active sites [36, 37], which severely hinders the rational optimizations of the Cu‐based ECO_2_R electrocatalysts. Against this backdrop, Cu‐based molecular hybrid materials, in which molecular catalysts like metal (e.g., Fe, Co, etc.) porphyrins or phthalocyanines are immobilized on a Cu‐derived surface, can offer distinct advantages in establishing a clear structure‐activity relationship, primarily due to their more definable, stable, and synthetically tunable molecular structures [38, 39, 40]. Moreover, in the context of the scarce strategies proposed to retain the Cu‐based catalyst structure during ECO_2_R [41, 42, 43], the strong binding of molecular catalysts can act as a potential method to circumvent the over‐reconstruction of the Cu substrate by utilizing the excess electrons in mediating CO_2_‐CO conversion (as discovered in this work); such electron utilizations can even further improve Cu‐*CO coverage [5, 44, 45, 46, 47, 48] to achieve the consecutive CO_2_‐CO and CO‐C_2+_ transformation in a cooperative manner. In light of the multiple merits of the Cu‐based molecular hybrid materials, as the proof of concept, we have designed three metal phthalocyanine (MPc)‐Cu_2_O hybrid electrocatalysts as the structurally defined models, aiming to bridge the gap between mechanistic findings and structure‐activity relationship in Cu‐catalyzed ECO_2_R. With the optimal metal‐ligand cooperativity, the covalently linked **NiPc‐OTh@Cu_2_O** (**NiPc‐OTh** = Ni(II) *β*‐tetrakis[2‐(3‐thienyl)ethoxy]phthalocyanine) displays the highest C_2+_ Faradaic efficiency (FE) in both H‐cells and gas‐diffused electrolyzers up to 78.3%, outperforming bare Cu_2_O, the non‐covalently assembled **NiPc@Cu_2_O** and its Co‐analog, **CoPc‐OTh@Cu_2_O** (**CoPc‐OTh** = Co(II) *β*‐tetrakis[2‐(3‐thienyl)ethoxy]phthalocyanine), and most documented Cu‐based molecular hybrid electrocatalysts. Interestingly, albeit with minor CO‐evolving capabilities when immobilized on multi‐walled carbon nanotubes (CNTs), the unexpectedly advantageous tandem catalysis in C_2+_ production with **NiPc‐OTh** over **CoPc‐OTh** when anchored on Cu_2_O has been noticed. In situ X‐ray absorption and in situ Raman spectroscopies demonstrate the highly stabilized Cu(I) configurations upon anchorage with the robust OTh‐appended MPc complexes during ECO_2_R, thereby retaining the well‐defined catalyst structures for clarifying structure‐activity relationships by further in situ spectroscopic and computational studies. The mechanistic insights indicate that the optimal Cu‐S‐Ni‐involved microenvironment within **NiPc‐OTh@Cu_2_O**, including the abundant Cu(I)Cu(0)‐paired active sites and the electronically optimal Ni center, collectively enables the most favorable kinetics in both the key CO‐transfer and *OCCO‐*OCCOH steps.

## 2 Materials and Methods

### 2.1 Synthesis of MPc‐OTh

4‐[2‐(3‐Thienyl)ethoxy]phthalonitrile was synthesized following a reported procedure with 4‐nitrophthalonitrile, 2‐(3‐thienyl)ethanol, and K_2_CO_3_ in dry DMSO under Ar atmosphere. The obtained intermediate was directly used for the preparation of metal phthalocyanines (MPc‐OTh, M = Ni, Co) via a conventional cyclotetramerization route using corresponding metal acetate hydrates and DBU in pentanol under reflux for 24 h. The products were purified by column chromatography (silica gel, CHCl_3_/CH_3_OH/THF). The obtained MPc‐OTh were characterized by UV–vis and HR‐MS, consistent with the expected molecular formulas.

### 2.2 Electrochemical Tests in H‐cell

ECO_2_R was performed in a gas‐tight H‐cell separated by an Nafion 117 membrane, using a carbon fiber paper (CFP) loaded with catalyst ink (0.1 mg cm^−2^) as the working electrode, Ag/AgCl as the reference, and Pt foil as the counter electrode. The ink was prepared by dispersing 1 mg of catalyst in isopropanol/Nafion solution (950:50 µL) via sonication. All tests were carried out in 0.5 M KHCO_3_ saturated with CO_2_ (99.99%) without gas bubbling during electrolysis. Potentials were converted to the RHE scale with 85% iR compensation.

### 2.3 Electrochemical Tests in Flow‐Cell

Flow‐cell experiments were performed in a three‐compartment reactor equipped with gas, cathode, and anode chambers (Gauss Union Co., Ltd.). Catalyst‐coated gas diffusion electrodes (GDEs, 0.5 mg cm^−2^) were prepared by drop‐casting the catalyst ink onto PTFE‐treated carbon paper. CO_2_ (30 mL min^−1^) and 1 M KOH electrolyte (15 mL min^−1^) were continuously supplied to the cathode chamber. A platinum foil and Ag/AgCl electrode served as counter and reference electrodes, respectively. Potentials were converted to RHE with 85% iR correction.

### 2.4 Product Quantification

Gas products (H_2_, CO, CH_4_, C_2_H_4_) were analyzed by gas chromatography (GC9790Plus, FULI INSTRUMENTS) equipped with TCD and FID detectors using Ar as carrier gas. Liquid products (HCOO^−^, CH_3_COO^−^, C_2_H_5_OH, isopropanol) were quantified by ^1^H NMR using D_2_O with DMSO as an internal standard. Product concentrations were determined by integrating characteristic proton signals relative to DMSO. Detailed analytical procedures are described in the Supplementary Material.

More detailed materials and methods can be found in the Supplementary Material, including sample synthesis, structural characterizations, properties measurements, and computational methods.

## 3 Results

### 3.1 Immobilizations on CNTs for ECO_2_R to CO

A comprehensive immobilization scenario was expected to ensure the well‐defined microenvironment between the Cu surface and the immobilized molecular catalysts in addition to their clear structures. In contrast to most documented Cu‐based molecular hybrid electrocatalysts constructed via weak van der Waals interactions [5, 45, 46], we deliberately install the thiophene pendants on the MPc backbone for covalent Cu‐S bonding at the Cu‐based materials, presumably enhancing the anchoring stability for a relatively fixed microenvironment, which also avoids possible molecule aggregation from the self‐polymerization by using thiol anchors in a recent case [37]. Accordingly, **NiPc‐OTh** and **CoPc‐OTh** were synthesized by two‐step reactions (see Supporting Information (SI) for details) and subsequently characterized by elemental analysis, UV‐vis absorption spectroscopy (Figures S1 and S2), high‐resolution mass spectroscopy (Figures S3 and S4), and cyclic voltammetry in homogeneous phase (Figures S5 and S6).

Subsequently, **NiPc‐OTh** and **CoPc‐OTh**, with pristine Ni phthalocyanine (**NiPc**) for primary comparison, were homogeneously immobilized on CNTs as **NiPc‐OTh@CNT**, **CoPc‐OTh@CNT,** and **NiPc@CNT** molecular hybrids (see SI and Figures 1A, S7–S9 for details), respectively. Then they were dispersed on CFPs for evaluation of ECO_2_R to CO in 0.5 M KHCO_3_ aqueous solutions in a gas‐tight H‐cell. Initially, linear scanning voltammetry (LSV) curves under CO_2_ show that **CoPc‐OTh@CNT** exhibits the highest current density and the lowest onset potential among the three molecular hybrids (Figure 1B), suggesting its superior activity for ECO_2_R. Meanwhile, chronoamperometry measurements were carried out at different potentials with product detection. Only CO and H_2_ were detected by gas chromatography, and negligible liquid products were found by ^1^H NMR spectroscopy. The catalytic currents during electrolysis were relatively sustained for all three electrocatalysts (Figure S10). We found that the Faradaic efficiency for CO production (FE_CO_) of **NiPc‐OTh@CNT** or **CoPc‐OTh@CNT** is over 90% at a potential more negative than −0.65 V versus the reversible hydrogen electrode (RHE; all potentials throughout this work are referenced to RHE unless otherwise stated). while the FE_CO_ of **NiPc@CNT** demands the applied potential to be as negative as −0.82 V to surpass 90% (Figure 1C). Based on the FE values, the partial current densities for CO production (*j*_CO_) of **NiPc@CNT**, **NiPc‐OTh@CNT**, and **CoPc‐OTh@CNT** were calculated (Figure  1D; see SI for details). **CoPc‐OTh@CNT** exhibited the largest *j*_CO_ of 31.8 mA cm^−2^ at −0.77 V, higher than those of **NiPc‐OTh@CNT** (25.8 mA cm^−2^, −0.80 V) and **NiPc@CNT** (13.0 mA cm^−2^, −0.86 V), respectively. These results demonstrate that **CoPc‐OTh@CNT** is slightly more efficient than **NiPc‐OTh@CNT** in terms of *j*_CO_, while both of which substantially outperform the reference **NiPc@CNT**, attributable to several merits from the alkyl‐dangled thiophene groups, like their electron‐donating properties to stabilize the catalyst [49], steric hindrance to suppress the phthalocyanine aggregation [50, 51], hydrophobic environment to better access CO_2_ over H^+^ [38, 50], etc. Consequently, both the thiophene‐functionalized MPc complexes hold great promise as CO‐evolving molecular sites for subsequent tandem catalysis in Cu‐mediated ECO_2_R to C_2+_ products.

**FIGURE 1:**
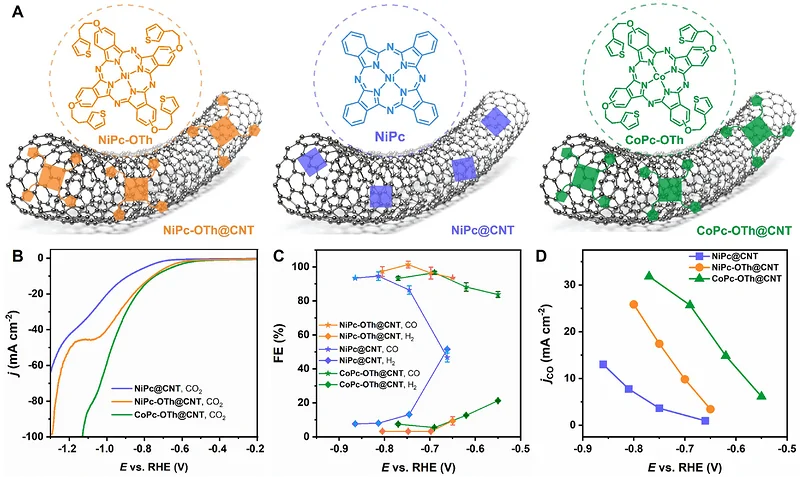
ECO_2_R to CO. (A) Chemical structures of **NiPc‐OTh**, **NiPc,** and **CoPc‐OTh** as well as their immobilizations on CNTs. (B) LSVs, (C) FE values and (D) CO partial current density of **NiPc‐OTh@CNT** (orange), **NiPc@CNT** (blue), and **CoPc‐OTh@CNT** (green) for ECO_2_R to CO in 0.5 M KHCO_3_.

### 3.2 Interactions Between the MPc Complexes and Cu_2_O

Cu_2_O nanocubes with lengths around 70 nm, expected as good prototypes for molecular immobilization, were prepared by a solution‐precipitation method (see SI for details) and then characterized by powder X‐ray diffraction (PXRD; Figure S11) and transmission electron microscopy (TEM; Figure S12). Besides their good catalytic performances for ECO_2_R to C_2+_ products, the main merit is their dominantly exposed {111} crystal face, which has also been found [25] to be relatively resistant to reconstruction during ECO_2_R, thereby improving the ease and compatibility of the subsequent computational modelling. Later, to construct the molecule‐Cu interfaces, the Cu_2_O nanocubes were mixed with the three MPc complexes in tetrahydrofuran (THF) to get homogeneous ink. Interestingly, we observed a stronger aggregation tendency with increasing concentrations of **NiPc‐OTh** in the Cu_2_O suspension, which tends to form bulky green particles upon sonication at [**NiPc‐OTh**] ≥ 0.15 mM, while no similar phenomena took place in the case of **NiPc** (Figure S13). In parallel mixing, **CoPc‐OTh** also induced the aggregation of Cu_2_O nanocubes to form small clusters, but to a much lesser extent than **NiPc‐OTh**. These observations imply the strongest binding affinity of **NiPc‐OTh** on Cu_2_O surface, which was further indicated by the UV‐vis absorption spectra of the supernatant from the centrifuged inks, where that of **NiPc‐OTh@Cu_2_O** showed approximately 95% of the absorption intensity attenuated after Cu_2_O adsorption. In contrast, **CoPc‐OTh@Cu_2_O** showed a moderate attenuation of 65%, and **NiPc@Cu_2_O** showed only 50% reduction (Figures S14 and S15). These results indicate the varying interaction strengths between the MPc complexes and Cu_2_O nanocubes, where **NiPc‐OTh** exhibits the strongest binding affinity, followed by **CoPc‐OTh** and **NiPc** in sequence. After immobilization, the transmission electron microscopy (TEM) images of as‐prepared **NiPc@Cu_2_O, NiPc‐OTh@Cu_2_O,** and **CoPc‐OTh@Cu_2_O** reveal the intact size and shape of the Cu_2_O nanocubes, and its elemental mapping shows the homogeneous distribution of Ni/Co element (Figures S16–S18), confirming that the MPc complexes could be attached on the Cu_2_O surface without altering the nano‐cubic morphology.

To further investigate the Cu‐molecule interactions, X‐ray photoelectron spectroscopy (XPS) was employed to analyze the chemical states of Cu_2_O and the three molecular hybrid materials. As shown in the XPS spectra, no significant shift in the Cu(I) peak was observed before and after loading the MPc complexes, indicative of the unaltered bulk +1 oxidation state of Cu (Figure S19 and Table S1). However, the Ni 2p XPS peak exhibited a positive shift of approximately 0.60 eV upon grafting **NiPc‐OTh** onto Cu_2_O, while a similar shift of ∼0.60 eV was observed in the S 2p XPS spectrum (Figures 2A and 2B; detailed data in Figures S20–S21 and Tables S2 and S3). These observations suggest that the electron density of **NiPc‐OTh** is substantially extracted by the immobilization of Cu_2_O, rendering their strong electron communication between **NiPc‐OTh** and Cu_2_O. In contrast, **CoPc‐OTh** displayed a shift of approximately 0.24 eV in both the Co 2p and S 2p spectra, indicating a moderate interaction between **CoPc‐OTh** and Cu_2_O, while weaker than that of **NiPc‐OTh@Cu_2_O** (Figures S22 and S23 and Tables S3 and S4). For **NiPc**, no significant XPS peak shift (≤ 0.10 eV) was observed before and after loading onto Cu_2_O (Figure S24 and Table S2), suggesting a much weaker Cu‐molecule interaction. These XPS results support the above synthetic observations related to the different strengths of the Cu‐molecule interactions between the MPc complexes and Cu_2_O, which vary highly along with both the substituents and the metal centers.

**FIGURE 2:**
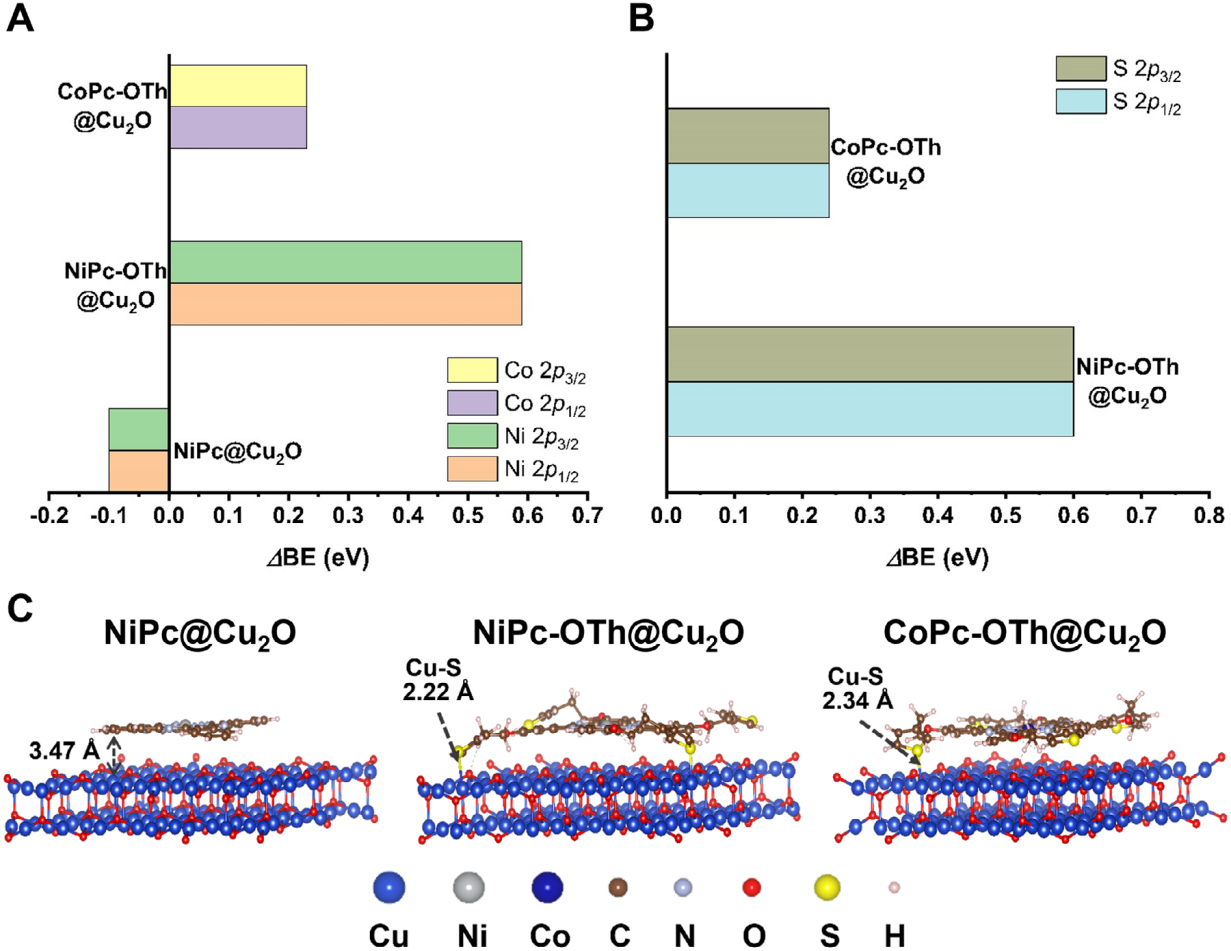
Cu‐molecule interactions. The binding energy shifts (Δ BE) of (A) Ni 2p and Co 2p, or (B) S 2p signals (except **NiPc**) in the XPS spectra of the MPc complexes (**NiPc**, **NiPc‐OTh,** or **CoPc‐OTh**) before and after immobilization on Cu_2_O. (C) Side view of the calculated structures of **NiPc@Cu_2_O**, **NiPc‐OTh@Cu_2_O,** and **CoPc‐OTh@Cu_2_O**.

To shed light on the nature of Cu‐molecule interactions, DFT calculations were performed to fabricate the molecular models of the MPc complexes immobilized at the {111} face of Cu_2_O crystal, along with the calculation of the adsorption energies (Δ*E*_ads_; see SI for calculation details). The results show that **NiPc**, **NiPc‐OTh,** and **CoPc‐OTh** all exhibit negative Δ*E*_ads_ values, confirming that their binding to Cu_2_O is thermodynamically favorable and thus spontaneous. Notably, the Δ*E*_ads_ values follow an order of **NiPc‐OTh** > **CoPc‐OTh** > **NiPc**, indicating that **NiPc‐OTh** exhibits the strongest adsorption with Cu_2_O. This trend is highly consistent with the XPS results as well as our visual and UV‐vis absorption spectral observations. Indeed, the dangling thiophene groups in **NiPc‐OTh** form two Cu‐S bonds with lengths of 2.71 and 2.22 Å, resulting in stronger chemical adsorption and thus a lower Δ*E*_ads_ (Figure 2C and Table S5). In contrast, **CoPc‐OTh** forms only one Cu‐S bond (2.34 Å), leading to weaker interactions compared to those in **NiPc‐OTh@Cu_2_O**. This difference may be attributed to the more *d*‐electron‐deficient nature of the Co center, which makes the corresponding thiophene pendents less coordinative [40, 52]. Last, the **NiPc**‐Cu_2_O interaction is primarily driven by van der Waals forces rather than covalent linkages, with no significant chemical bonding.

### 3.3 ECO_2_R to Multicabon Products

With understanding of the substituent‐ and metal‐sensitive Cu‐molecule interactions, the ECO_2_R activity of these Cu‐based molecular hybrid materials was evaluated in CO_2_‐saturated 0.1 M KHCO_3_ aqueous solutions in an H‐cell setup. The working electrodes were made by drop‐casting the as‐prepared THF inks on the CFPs, which were then subjected in control‐potential electrolysis (CPE; see SI for details). Among the examined applied potentials with the optimal loading (See SI Figure S25 for details), **NiPc‐OTh@Cu_2_O** displays a much‐improved FE for C_2+_ products, including ethylene, ethanol, acetate, and 1‐propanol, relative to the bare Cu_2_O (Figure 3A, Figure S26, and Table S6), while **NiPc@Cu_2_O** shows decreased $FE_{C_{2+}}$ and exacerbated H_2_ evolution. In particular, at ‐1.04 V, **NiPc‐OTh@Cu_2_O** afforded $FE_{C_{2}H_{4}}$ of 49 ± 2% and $FE_{C_{2+}}$ of 68 ± 1%, far surpassing those from the bare Cu_2_O (26 ± 2% and 37 ± 5%) and **NiPc@Cu_2_O** (25 ± 1% and 26 ± 2%), respectively. Although **NiPc‐OTh** has exhibited higher performance than **NiPc** in ECO_2_R to CO when immobilized on CNTs, their $FE_{\mathrm{CO}}$ values are similarly over 90% at potentials more negative than ‐0.8 V (Figure 1), still more positive than the applied potentials for Cu‐catalyzed ECO_2_R to C_2+_ products (−0.96 ∼ −1.08 V). This means that the thermodynamic disadvantage of **NiPc** in CO_2_‐CO conversion could be less influential in the C_2+_‐production system, which in turn highlights the key contribution of MPc‐Cu interactions in improving the C_2+_ selectivity in the case of **NiPc‐OTh@Cu_2_O**.

**FIGURE 3:**
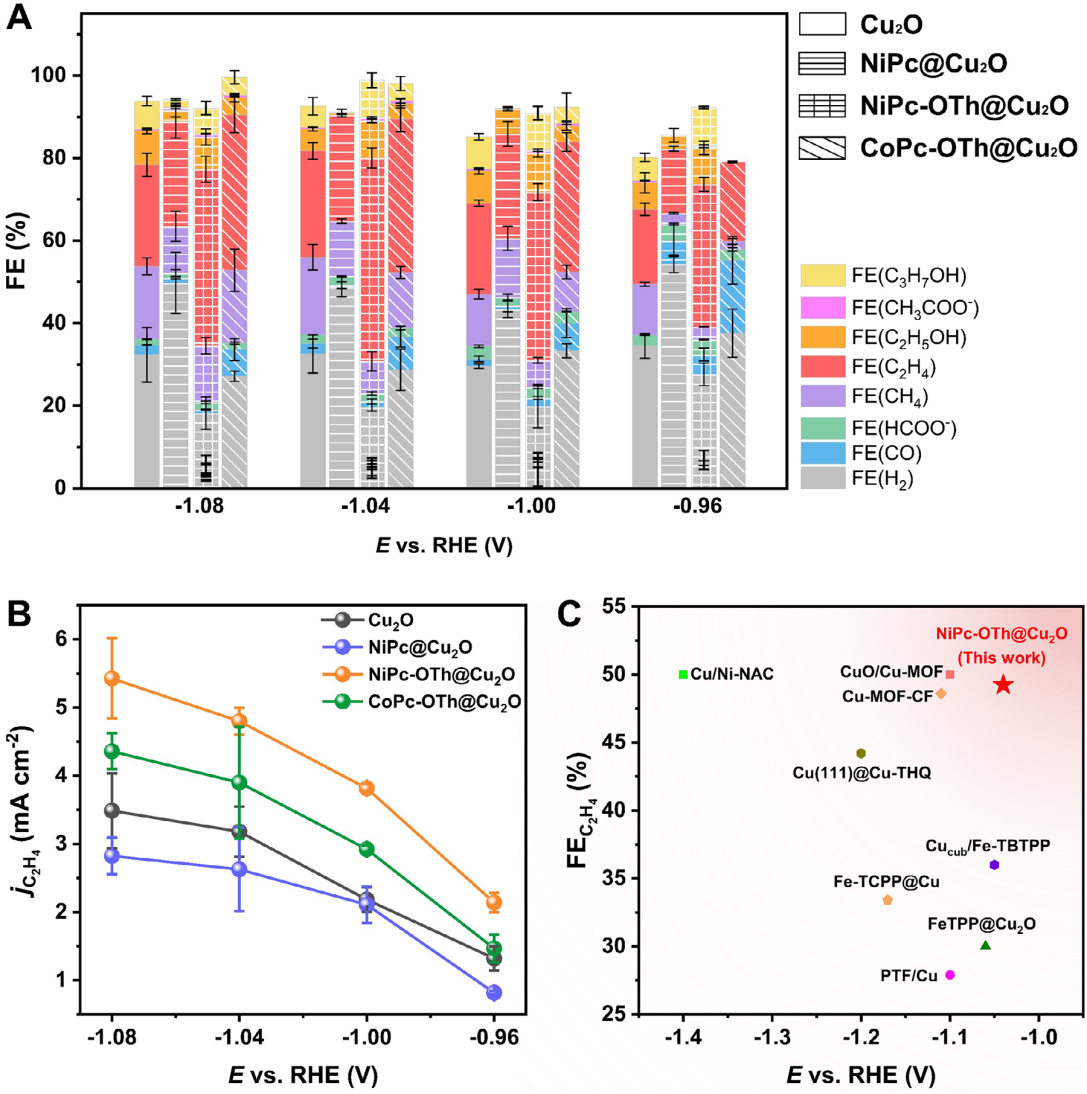
ECO_2_R to C_2+_. (A) Potential‐dependent performances in ECO_2_R with bars indicating FEs for bare Cu_2_O, **NiPc@Cu_2_O**, **NiPc‐OTh@Cu_2_O** and **CoPc‐OTh@Cu_2_O** in a gas‐tight, H‐cell set‐up. The loading of metal complex was set at 10 nmol cm^−2^. (B) Partial current densities of C_2_H_4_ production. (C) Comparison of the $FE_{C_{2}H_{4}}$ values of **NiPc‐OTh@Cu_2_O** and other representative Cu‐based tandem catalysts.

Interestingly, **CoPc‐OTh**, which shares the same thiophene‐based substituents and exhibits better CO_2_‐CO performance than **NiPc‐OTh**, did not show superior C_2+_ selectivity. At ‐1.04 V, the $FE_{C_{2}H_{4}}$ of **CoPc‐OTh@Cu_2_O** was 37.2 ± 3.00%, showing only a slight improvement over Cu_2_O. In addition, compared to Cu_2_O (2.52 ± 1.05%), **CoPc‐OTh@Cu_2_O** (8.03 ± 2.52%) exhibited an increased FE_CO_. This suggests that the CO produced by **CoPc‐OTh** was not effectively captured by the Cu_2_O surface to participate in C‐C coupling reactions. This indicates that, aside from the thiophene‐Cu interactions, the metal center also plays a critical role in tuning C_2+_ production. Moreover, **NiPc‐OTh@Cu_2_O** exhibited the highest partial current densities for C_2_H_4_ production ($j_{C_{2}H_{4}}$; Figure 3B) of 4.8 mA cm^−2^ at −1.04 V, which is 1.26, 1.85, and 1.55 times higher than those of **CoPc‐OTh@Cu_2_O** (3.8 mA cm^−2^), **NiPc@Cu_2_O** (2.6 mA cm^−2^) and Cu_2_O only (3.1 mA cm^−2^), respectively. To the best of our knowledge, the $FE_{C_{2}H_{4}}$ value and especially the corresponding applied potential of **NiPc‐OTh@Cu_2_O** outperform most reported Cu‐based, noble‐metal‐free catalysts for ECO_2_R (Figure 3C and Table S7) [45, 46, 53, 54, 55, 56, 57]. The TEM analysis showed that after electrolysis, the Cu_2_O nanocubes became spongy with retention of the cube‐like shape and the cluster‐free dispersion of Co/Ni elements (Figures S27–S29), which suggests the limited reconstruction of the Cu‐based nanoparticles [19, 58] and the intact feature of the immobilized MPc complexes.

Encouraged by the high performance of **NiPc‐OTh@Cu_2_O** in the H‐cell setup, we further fabricated gas‐diffusion electrodes (GDEs) to pursue industrial‐relevant efficiency with its improved mass transport [49, 59, 60, 61, 62, 63]. To construct the GDEs, **NiPc‐OTh@Cu_2_O** was deposited onto a microporous gas diffusion layer (GDL) via drop‐casting, achieving a catalyst loading of 0.5 mg cm^−2^ (see SI for details). The electrolysis was performed in flowing 1.0 M KOH electrolyte. The **NiPc‐OTh@Cu_2_O**‐based GDE demonstrates high current densities (>200 mA cm^−2^) at moderate overpotentials (−0.9, −0.96, −1.06, −1.12 V), along with excellent FE for C_2+_ products (>63%). Notably, at −0.96 V, the catalyst achieved an FE of 60 ± 4.5% for ethylene and 78 ± 1.3% for C_2+_ products at a current density of ∼330 mA cm^−2^, respectively, demonstrating the excellent catalytic performance of **NiPc‐OTh@Cu_2_O** for CO_2_‐C_2+_ conversion (Figures S30 and S31). Next, we tested the energy efficiency of the GDE electrolyzer under these conditions. The results showed that at ‐0.96 V, with a total cell voltage of 4.4 V, the cathodic energy efficiency for C_2+_ products reached 41.1 ± 0.7%, and the full‐cell energy efficiency was 20.6%. Given these performances, this strategy appears to be highly promising for the practical application of converting CO_2_ into high‐value‐added chemicals using renewable electricity.

### 3.4 In‐Situ X‐Ray Absorption Fine Structure (XAFS)

The above performances demonstrate the key role of the metal‐ligand cooperativity in the Cu‐based molecular hybrid electrocatalysts. Specifically, in contrast to the mostly reported effects of ligand structure, it is interesting to notice the unexpected effect of metal centers in this case. To elucidate the structure‐activity relationship, a sequence of in situ spectroscopies was implemented with XAFS as the beginning to monitor the dynamic changes in local atomic and electronic structures of the **NiPc@Cu_2_O**, **NiPc‐OTh@Cu_2_O,** and **CoPc‐OTh@Cu_2_O** hybrid materials. Figures 4A and 4E present the X‐ray absorption near‐edge structure (XANES) spectra in Ni K‐edge of **NiPc‐OTh@Cu_2_O** and **NiPc@Cu_2_O**, which are essentially the same under different applied potentials. Particularly, the peak shape and intensity of pre‐edge peaks at 8334.0 eV and shoulders at 8339.5 eV remain unchanged, which are assignable to the transitions of 1s → 3d/4p and 1s → 4p_z_, respectively, indicating the maintaining symmetry and planar feature around nickel centers in both catalysts, consistent with the TEM results (Figure S27 and S28).

**FIGURE 4:**
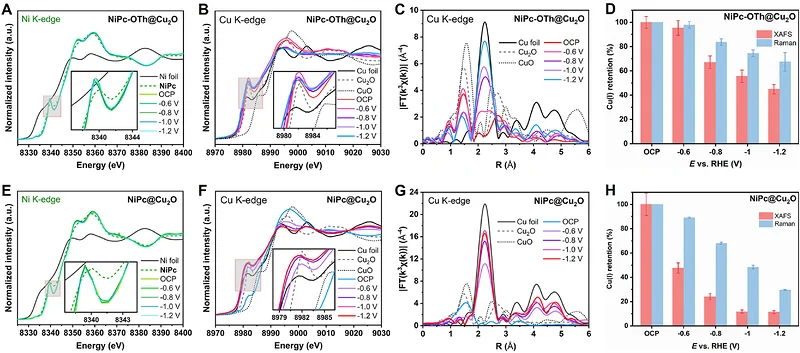
In situ XANES spectra of **NiPc‐OTh@Cu_2_O** or **NiPc@Cu_2_O** at Ni K‐edge (A, E) and Cu K‐edge (B, F) at different applied potentials. Insets in (A, B, E, F) provide magnified views. (C, G) *k* ^3^‐weight Cu K‐edge FT‐EXAFS spectra at different applied potentials. (D, H) Retention of Cu(I) species under different applied potentials, normalized to the one under OCP condition (set as 100%). Cu(I) fractions are derived from in situ XAFS (red) and in situ Raman (blue) measurements.

However, the local configuration of copper species of these two catalysts exhibits distinct evolution trends. As shown in Figures 4B and 4F, the XANES spectra in the Cu K‐edge of fresh **NiPc‐OTh@Cu_2_O** and **NiPc@Cu_2_O** show obvious shoulders at 8982.0 eV which is consistent with the reference Cu_2_O. Besides, the absorption edge in Cu K‐edge lies between Cu foil and CuO, further indicating the +1 oxidation state for these catalysts. Moreover, compared with bare Cu_2_O, the Fourier‐transformed extended X‐ray absorption fine structure (FT‐EXAFS) spectra of **NiPc‐OTh@Cu_2_O** and **NiPc@Cu_2_O** show a weaker intensity of the Cu‐O‐Cu scattering interaction peak at 2.7 Å, demonstrating the small particle size, which corresponds with the TEM result. By applying the open‐circuit potential (OCP), neither the FT‐EXAFS (Figure 4C and Figure S32) nor the XANES spectra exhibited any change for **NiPc‐OTh@Cu_2_O**. As shown in Figures 4C and 4G, the main difference in FT‐EXAFS of **NiPc‐OTh@Cu_2_O** and **NiPc@Cu_2_O** lies in the second coordination peak at ∼2.7 Å which corresponds to the Cu‐O‐Cu scattering path, indicating the electron transfer from Cu_2_O to **NiPc‐OTh** and corresponding to the fitting parameters of coordination number in Tables S8 and S10.

Under the ECO_2_R conditions, the shoulder peak position in the XANES spectrum of **NiPc‐OTh@Cu_2_O** shows no significant shift. However, a new satellite peak appeared and leftward shifted from the shoulder peak at 8980.0 eV, indicative of Cu(I)/Cu(0) reduction. With the potential of −0.6 V, a peak at 2.2 Å emerged at FT‐EXAFS spectra of **NiPc‐OTh@Cu_2_O** which is aligned with the main peak of Cu foil and can be assigned to the Cu‐Cu interaction path, indicating that Cu_2_O nanoparticles are gradually reduced to Cu(0) clusters. At potentials from ‐0.8 to −1.2 V, the intensity of the Cu‐Cu scattering peak gradually increases, while that of the Cu‐O‐Cu scattering peak progressively disappears. The Cu‐O scattering peak at ∼1.5 Å gradually decreased to substantial intensity. This indicates that under negative potentials, the catalyst evolves into a state mixed with metallic and oxidized particles. To obtain a quantitative coordination environment, the least‐square fitting of FT‐EXAFS was performed. As displayed in Table S8, the coordination number of Cu‐Cu scattering path increases from 0.9 to 4.0 at the potential range of −0.6 to −1.2 V, demonstrating the small size of Cu clusters. Similarly, as shown in Figure S33, S34, and Table S9, for **CoPc‐OTh@Cu_2_O** at −1.2 V, the Co K‐edge XAFS remains unchanged, and the Cu K‐edge XAFS spectra shows largely retained Cu‐O and Cu‐O‐Cu scattering peaks, further indicating the Cu(I) substantially stabilized by the OTh‐anchored MPc complexes.

In contrast, in the case of **NiPc@Cu_2_O**, as the negative potential was applied, the shoulder peak in the XANES spectrum shifted rapidly to the left and aligned with that of Cu foil. Also, the position of the whiteline peak is almost the same as that of Cu foil, suggesting the dominant reduction to Cu(0). The changes in FT‐EXAFS spectra also illustrate that the Cu_2_O nanoparticles were fully reduced to Cu(0) at negative potentials (Figure 4G and Figure S35). As shown in Table S10, the coordination number of Cu‐Cu interaction is about 7 at potentials more negative than ‐1.0 V, indicating the size of copper particles gradually stabilizes and remains relatively large.

The above differences in the evolution of the copper speciation can be more visible in Figures 4D and 4H (with details in Figures S36 and S37), where the incorporation of the ‐OTh group in **NiPc‐OTh@Cu_2_O** and **CoPc‐OTh@Cu_2_O** effectively stabilizes the Cu(I) oxidation state, while Cu_2_O tends to be reduced to Cu(0) nanoparticles in **NiPc@Cu_2_O** under ECO_2_R conditions. The substantial *operando* retention of Cu(I) centers in the former cases suggests not only the less reconstructed Cu component but also the facile formation of Cu(I)Cu(0) diatomic sites, which can steer selectivity toward C_2_ products compared to bare Cu(I) or Cu(0) atoms [64, 65, 66, 67, 68, 69, 70, 71, 72, 73, 74, 75]. Such strong Cu(I) stabilization should originate from the facile interfacial charge transport via the strong Cu‐molecule interactions endowed by the 2‐(3‐thienyl)ethoxy anchors (see Section 3.2). Consequently, the suitable immobilization of molecular catalysts on the Cu‐based materials can be an advantageous strategy to stabilize the Cu electrocatalysts and also improve the performance.

### 3.5 In Situ Vibrational Spectroscopies

To further verify the structure‐activity relationship of the three MPc@Cu_2_O hybrid electrocatalysts in ECO_2_R‐C_2+_ conversion, we examined the possible adsorbed intermediates by combining in situ Raman and Fourier‐transformed infra‐red (FT‐IR) spectroscopies. Initially, the in situ Raman spectroscopy was coupled with electrochemical analysis for the three Cu‐based molecular hybrid materials under 1 atm CO_2_ in 0.1 M KHCO_3_. The results are shown in Figures 5A–C, with the assignments of the detected Raman‐active species in Table S11. The prior comparison focuses on the effects of the thiophene anchors. First, the characteristic Cu(I) Raman peaks appear in the 140‐230 and 520‐630 cm^−1^ [19, 47, 76]. For **NiPc@Cu_2_O**, this signal was weakened significantly at potentials more negative than ‐0.6 V, suggesting the reduction of Cu_2_O to metallic Cu. However, for **NiPc‐OTh@Cu_2_O** and **CoPc‐OTh@Cu_2_O**, both of their Cu(I) characteristic peaks were largely retained, consistent with the in situ XAFS results (Figures 4D and 4H).

**FIGURE 5:**
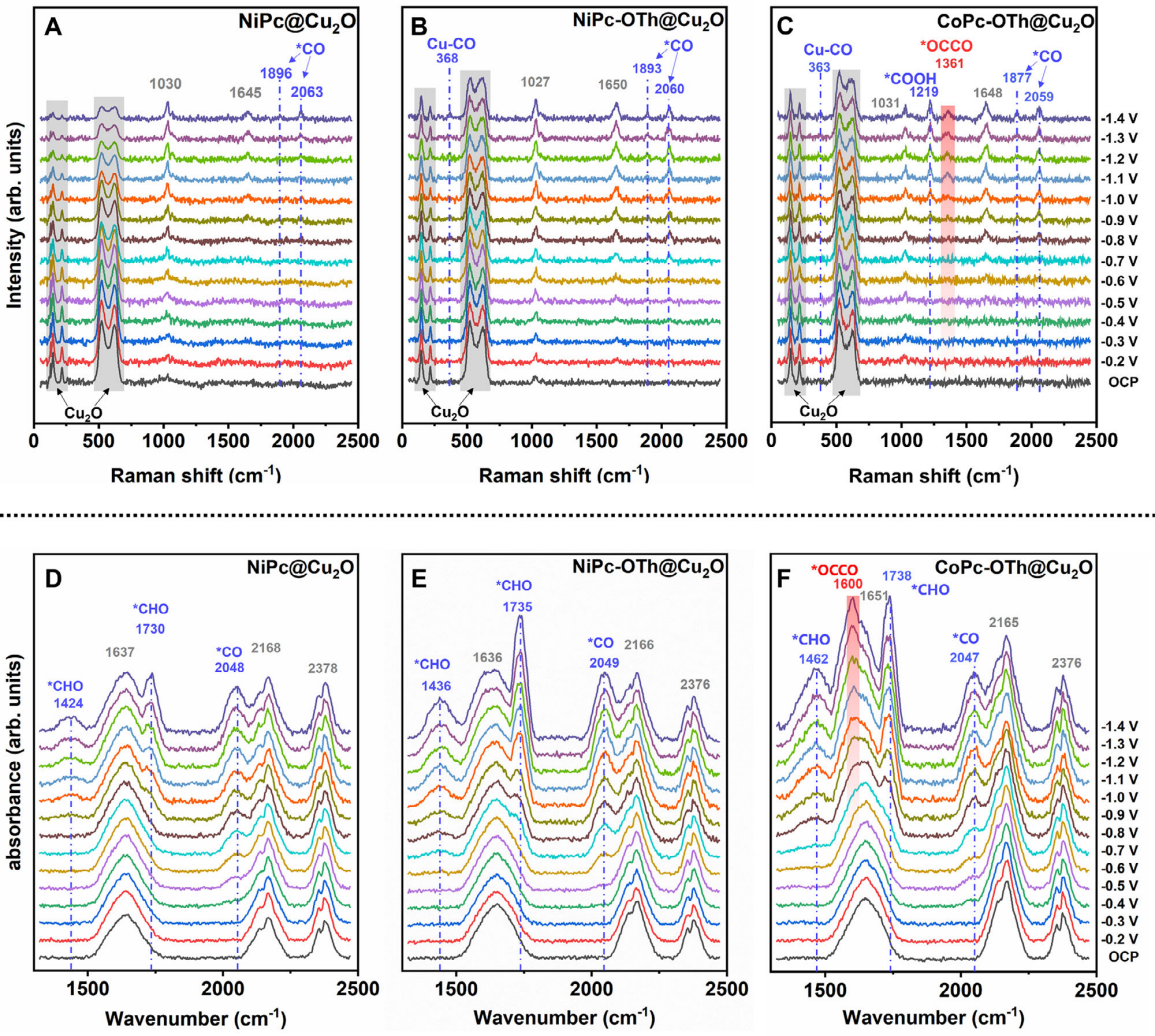
In situ Raman spectroscopies of (A) **NiPc@Cu_2_O**, (B) **NiPc‐OTh@Cu_2_O,** and (C) **CoPc‐OTh@Cu_2_O**, and in situ FT‐IR spectroscopies of (D) **NiPc@Cu_2_O**, (E) **NiPc‐OTh@Cu_2_O,** and (F) **CoPc‐OTh@Cu_2_O**, all measured in 0.1 M KHCO_3_ under CO_2_ saturation at varying applied potentials.

Besides the varied Cu oxidation states, further insights are revealed by comparative analysis on the CO‐bound intermediates from the in situ Raman and subsequently in situ FT‐IR spectroscopy. On one hand, the Cu‐CO vibrations at 270 and 350 cm^−1^, as well as the C‐O stretching signals of *CO at 1850 and 2000 cm^−1^ [19, 25, 47, 76, 77], could be observed for all samples. More specifically, these *CO‐related signals appeared with higher intensities at potentials < ‐0.6 V in the cases of **NiPc‐OTh@Cu_2_O** and **CoPc‐OTh@Cu_2_O** over those of **NiPc@Cu_2_O**. Accordingly, the in situ FT‐IR spectroscopic outcome (Figures 5D–F with signal assignments in Table S12) also displays that the **NiPc‐OTh@Cu_2_O** and **CoPc‐OTh@Cu_2_O** systems exhibit stronger signals for the *CHO (1420, 1730 cm^−1^) [78, 79, 80] and *CO (2045 cm^−1^) [78, 81, 82] intermediates. The *CHO species may possibly be hydrogenated to form methane or couple with *CO to generate *OCCHO, an intermediate toward ethylene generation [83, 84]. Presumably, these intensity differences in CO adsorption during electrocatalysis should originate from the use of thiophene‐appended MPc complexes, which, compared to the pristine **NiPc**, not only possess the enhanced CO‐evolution capability but also pose close molecule‐Cu interactions for easy CO transfer to the Cu surface [37], thereby promoting C_2+_ product formation in a cascade manner.

On the other hand, the differences in the *C_2_‐derived intermediates, which also govern the C‐C coupling efficiency, were noticed in the in situ vibrational spectroscopies, unexpectedly dependent on the different MPc metal centers. That is, unlike **NiPc‐OTh@Cu_2_O**, the in situ Raman spectra of **CoPc‐OTh@Cu_2_O** exhibit additional peaks at 1219 and 1361 cm^−1^ (Figures 5B and 5C), assignable to the *COOH and *OCCO intermediates, respectively. The *COOH intermediate has been detected and proposed as the precursor of *CO species at 1200–1300 cm^−1^ (Table S13) [85, 86, 87, 88, 89, 90], reminiscent of the strongest CO‐evolution activity of **CoPc‐OTh@Cu_2_O** (Figure 1D). This attribution has also been confirmed with a main peak at 1248 cm^−1^ in its DFT‐simulated Raman spectrum (Figure S38). More importantly, in the context of the much less documented and more varied reported Raman signals (Table S14) [25, 86, 90, 91, 92], the *OCCO species was computationally verified herein within the **CoPc‐OTh@Cu_2_O** model (main signal at 1337 cm^−1^; Figure S39). Further evidence comes from the in situ FT‐IR spectra of **CoPc‐OTh@Cu_2_O**, where a spectral evolution at 1600 cm^−1^ was not observed in the parallel spectra with **NiPc‐OTh@Cu_2_O** or **NiPc@Cu_2_O**; this signal can also be assigned to the *OCCO intermediate based on our DFT simulations (main signal at 1576 cm^−1^; Figure S40), also in agreement with the reported *OCCO FT‐IR signals [25, 90]. Given that the observations of the *OCCO intermediate demand a substantial accumulation to a detectable concentration, their spectral evolution in the above in situ vibrational spectroscopies in turn suggests that **CoPc‐OTh@Cu_2_O** may provide a Co‐involved microenvironment unfavorable to the conversion of *OCCO species into further *C_2_ intermediates and consequently the final C_2+_ products.

### 3.6 Energy Diagrams From DFT Calculations

To further elucidate the structure‐activity relationship in this family of Cu‐based molecular hybrid electrocatalysts, DFT calculations were performed to offer the energy diagram of two key processes, CO transfer and *OCCO‐*OCCOH conversion (an overall diagram in Figure S41; see Supplementary Material for calculation details), on the basis of the above detected adsorbates (Figure 5). On the one hand, the energy diagram for the CO transfer (Figure 6A and Figure S42) first indicates that the free energy of *CO at the MPc complexes follows an order of **CoPc‐OTh@Cu_2_O** (−1.18 eV) > **NiPc‐OTh@Cu_2_O** (−1.32 eV) > **NiPc@Cu_2_O** (−1.58 eV). This trend also corresponds to the activity differences of their CNT‐based hybrid electrocatalysts in CO_2_‐CO conversion (Figure 1), suggesting that the CO desorption may serve as the rate‐determining step. This trend is explained by the analyses of projected crystal orbital Hamilton population (pCOHP; Figure S43 with detailed discussion) and the partial density of states (PDOS; Figure S44 with detailed discussion) both on the *d* bands of the MPc complexes, where the electronic structures of the MPc complexes are found to be essential for their CO‐binding strengths.

**FIGURE 6:**
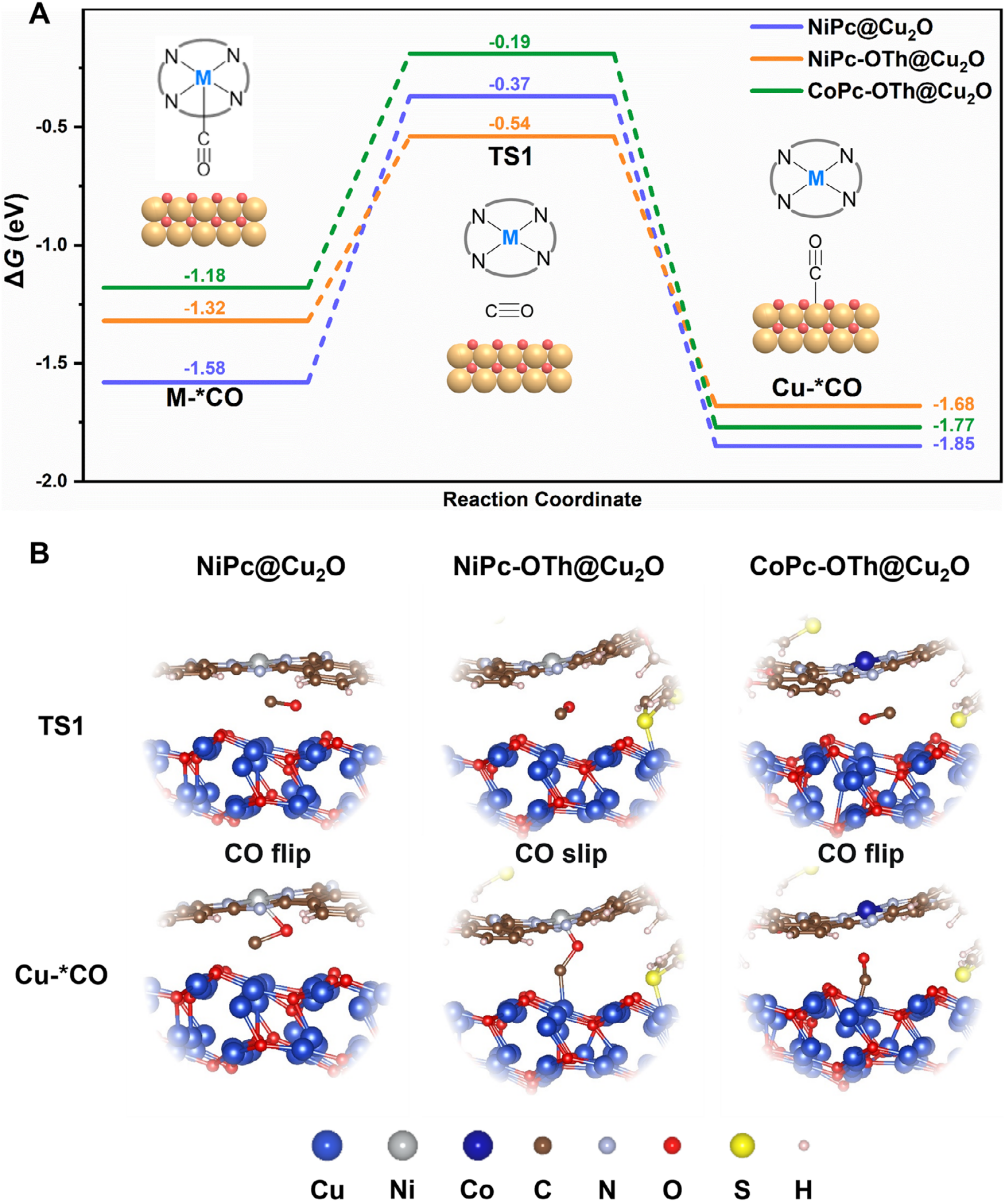
DFT‐calculated CO‐transfer mechanism. (a) Calculated free energy diagrams for *CO transfer in **NiPc@Cu_2_O**, **NiPc‐OTh@Cu_2_O**, and **CoPc‐OTh@Cu_2_O** during ECO_2_R. (b) Side view of the CO transfer transition state and Cu‐*CO intermediate in **NiPc@Cu_2_O**, **NiPc‐OTh@Cu_2_O**, and **CoPc‐OTh@Cu_2_O**.

More importantly, the CO‐transfer energy diagram indicates that each CO transfer from MPc to Cu_2_O is thermodynamically favorable, implying that the reaction would be dominated by its kinetics. Accordingly, distinct energy barriers for the transition from *CO to the first transition state (TS_1_) were obtained with the order of **NiPc@Cu_2_O** (1.21 eV) > **CoPc‐OTh@Cu_2_O** (0.99 eV) > **NiPc‐OTh@Cu_2_O** (0.78 eV), indicating that **NiPc‐OTh@Cu_2_O** enables the most efficient CO transfer, followed by **CoPc‐OTh@Cu_2_O**, with **NiPc@Cu_2_O** being the least favorable. Specifically, in the TS_1_ structure of **NiPc‐OTh@Cu_2_O**, the C atom of the CO molecule is attracted by the O atom of Cu_2_O, a configuration favorable to the slipping and final adsorption at the Cu_2_O surface; this advantageous geometry stabilizes the corresponding TS_1_ species. In contrast, the TS_1_ structure of either **NiPc@Cu_2_O** or **CoPc‐OTh@Cu_2_O** reveals a different configuration where CO is attracted by Cu_2_O at its O atom during the reaction, resulting in the additional CO‐flipping energy to form Cu‐CO species and thereby raising the TS_1_ barrier. During the flipping, specifically, CO is vertically adsorbed via its carbon atom on the Ni/Co site, and similarly, on the Cu_2_O surface, CO adopts a carbon‐down configuration. During this transfer process, the CO molecule undergoes a rotational reconfiguration, transitioning from a carbon‐up to a carbon‐down orientation, thereby enabling adsorption on the Cu_2_O surface in the energetically most favorable conformation. Furthermore, the overlapped density of states between the transferred CO molecule and the Cu_2_O surface (Figure S45) also suggests that the O atom on the Cu_2_O from the **NiPc‐OTh@Cu_2_O** model exhibits the largest overlap with the *p* orbital of CO among the three cases, which makes the energy change more continuous along the CO transfer process and therefore contributes to the lowest TS_1_ barrier.

After the formation of Cu‐CO intermediate, it couples with another CO to form the *OCCO intermediate (Figure S41), which was only detected in the **CoPc‐OTh@Cu_2_O** system Further DFT calculations were conducted to compare the reaction energetics of subsequent *OCCO‐*OCCOH conversions in the **NiPc@Cu_2_O**, **NiPc‐OTh@Cu_2_O,** and **CoPc‐OTh@Cu_2_O** systems. The *OCCO‐*OCCOH conversion are both thermodynamically favorable in all three catalysts (Figure 7), in turn suggesting that the kinetics of *OCCO‐*OCCOH conversion dominate the reaction progress. A closer look at the *OCCO‐*OCCOH conversion reveals that the hydrogen adsorption at the metal centers (Ni/Co in MPc or Cu sites, denoted as *OCCO‐H species; Figure 7A and Figure S46) is thermodynamically more favored than the direct hydrogenation of the *OCCO moiety; this suggests that the *OCCO‐H state can be the more direct reactant instead of *OCCO to yield the final *OCCOH species, making the TS2 barriers in the order of **NiPc@Cu_2_O** (1.35 eV) > **CoPc‐OTh@Cu_2_O** (0.89 eV) > **NiPc‐OTh@Cu_2_O** (0.51 eV). The origin of this trend was first revealed by the PDOS data of the MPc metal centers (Figure S44), which display the occupied states near the Fermi level for both **NiPc** and **NiPc‐OTh**, suggesting the favorable formation of Ni‐H species [93, 94]. This tendency is consistent with the optimized *OCCO‐H structure of either **NiPc@Cu_2_O** or **NiPc‐OTh@Cu_2_O**, where the Ni center serves as the most favorable H‐adsorption site (Figure 7B). Moreover, due to the electron‐donating effect of the thiophene groups, the Ni‐H bond in **NiPc‐OTh@Cu_2_O** is relatively weaker (**NiPc**: 1.15 Å; **NiPc‐OTh**: 1.20 Å; Figure S47), which further reduces the TS_2_ barrier of **NiPc‐OTh@Cu_2_O** for its easier hydrogen dissociation from the Ni center and then transfer to the OCCO moiety.

**FIGURE 7:**
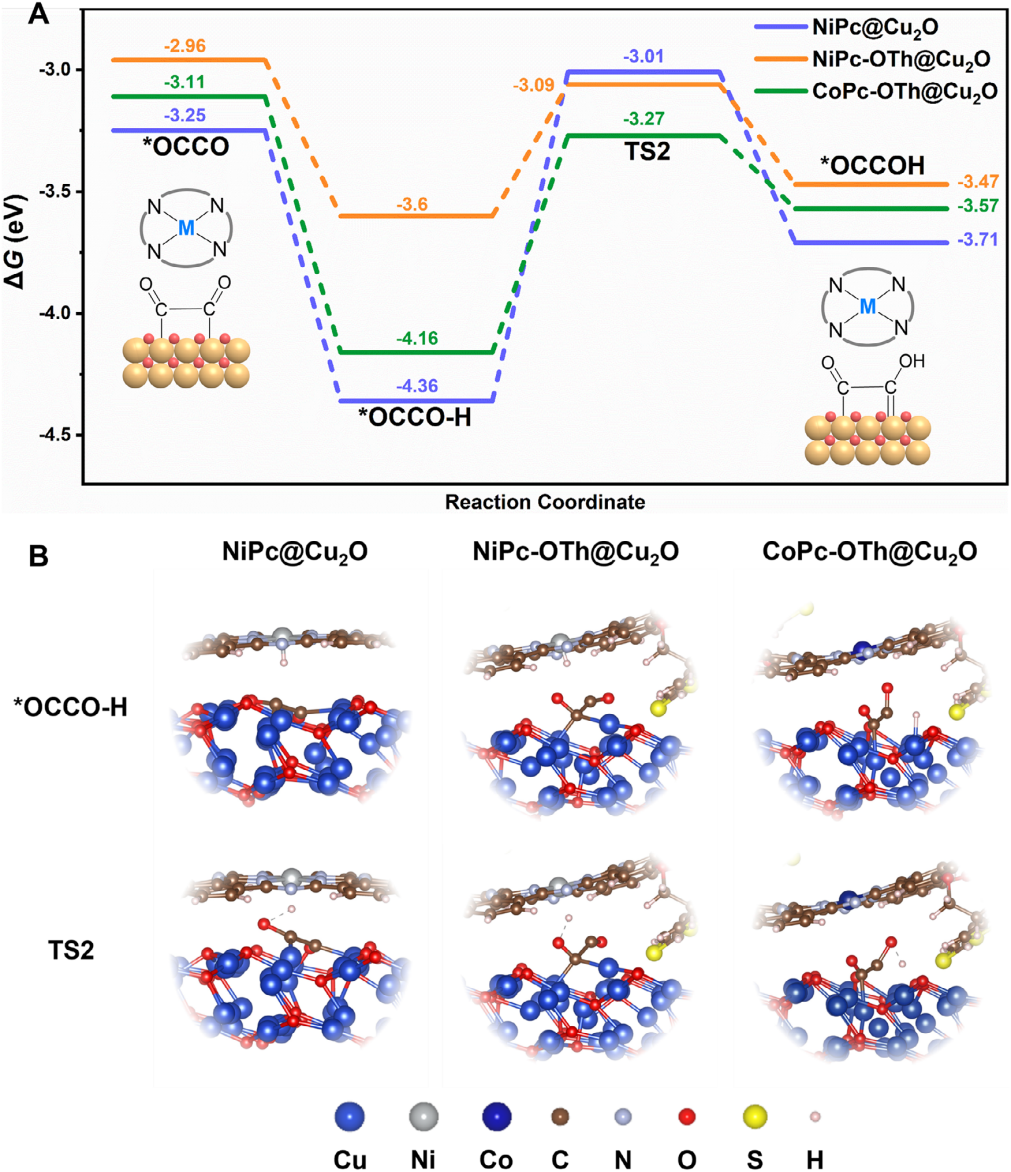
DFT‐calculated *OCCO‐*OCCOH‐conversion mechanism. (A) Calculated free energy diagram for the *OCCO‐*OCCOH conversion in **NiPc@Cu_2_O**, **NiPc‐OTh@Cu_2_O,** or **CoPc‐OTh@Cu_2_O** during ECO_2_R. (B) Side view of *OCCO‐H and TS2 structures of **NiPc@Cu_2_O**, **NiPc‐OTh@Cu_2_O** and **CoPc‐OTh@Cu_2_O**.

Interestingly, in the **CoPc‐OTh@Cu_2_O** system, the Co center tends not to adsorb hydrogen, as supported by the negligible state distribution near the Fermi level in the PDOS data of **CoPc‐OTh** (Figure S44), making the surface Cu atoms the more favorable H‐adsorption sites (Figure 7B). This preference is further corroborated by the much higher TS2 barrier with hydrogen adsorbed on the Co than the one on Cu (1.37 vs. 0.89 eV) (Figures S48 and S49). As a result, the hydrogen activation and migration to *OCCO predominantly occur via the Cu sites in the case of **CoPc‐OTh@Cu_2_O**. In this way, nonetheless, the hydrogen transfer from Cu to *OCCO inevitably follows a reaction scheme similar to that of the bare Cu_2_O surface, where the hydrogen transfer to the *OCCO moiety is hindered by the strong H‐attracting ability of the Cu_2_O surface [95, 96], causing a higher TS_2_ barrier; this induces the accumulation of the *OCCO species, which is thus detectable via the in situ vibrational spectroscopies (Figures 5C and 5F), eventually leading to a limited enhancement in C_2+_ production. Consequently, **NiPc‐OTh@Cu_2_O** possesses both the lowest TS_1_ and TS_2_ barriers relative to the other two, consistent with its highest performance in ECO_2_R.

## 4 Discussion

In this work, we employed Cu‐based molecular hybrid materials with well‐defined structures to investigate the structure‐activity relationship in the Cu‐catalyzed CO_2_‐C_2+_ conversion, which circumvents the mechanistic challenges induced by the ill‐defined and unstable structures of the extensively documented Cu‐based inorganic materials or coordination compounds [41, 42, 43]. After synthesis of the three MPc complexes with control over the metal centers and the thiophene pendants, their electrocatalytic performances toward ECO_2_R to CO upon anchorage on CNTs were initially evaluated. By comparing their FE_CO_ and *j*_CO_ values, **CoPc‐OTh@CNT** is the most active CO‐evolving electrocatalyst, and **NiPc‐OTh@CNT** is slightly less active, with **NiPc@CNT** being the least active one. Presumably, the highest CO‐desorption ability of the Co center, as indicated by DFT calculations (Figure 6A and Figures S43 and S44), as well as the favorable electronic [49] and steric effects [50, 51] of the thiophene groups, are both advantageous in improving the electrocatalytic performance of **CoPc‐OTh** as the intrinsic active sites of CO_2_‐CO conversion. However, different from the CO‐evolving activity trend among their CNT hybrids, the subsequent electrocatalytic evaluation for CO_2_‐C_2+_ conversion revealed that the more CO‐evolving‐active **CoPc‐OTh** could not substantially improve the C_2+_ production when immobilized on Cu_2_O nanocubes, with **NiPc** as the inactive CO enricher (Figures 1B–D, Figures 3A and 3B). In sharp contrast, **NiPc‐OTh@Cu_2_O** exhibits the highest $FE_{C_{2+}}$ of 78.3% and $j_{C_{2+}}$ values of 330 mA·cm^−2^ among these Cu_2_O‐based molecular hybrid materials, which are also comparable to those state‐of‐the‐art Cu‐based, noble‐metal‐free electrocatalysts under similar conditions.

Based on the above ECO_2_R performances, the structural‐activity relationship among the three molecular hybrid electrocatalysts was first revealed by their varied Cu‐molecule interactions, where **NiPc‐OTh** exhibits the highest **MPc‐Cu_2_O** affinity, followed by **CoPc‐OTh** and **NiPc**. This affinity order has been fully corroborated by visual aggregation tendency, UV‐vis monitoring of the mixture supernatant, and XPS and DFT modelling (Figure 2 and Figure S13–S15), demonstrating the reinforced electronic communications from non‐covalent interactions to covalent linkages by installing the thiophene anchors on the MPc complexes. Such facile interfacial electron delivery enables the substantial maintenance of Cu(I) species *except* in the prototypical **NiPc@Cu_2_O** system under ECO_2_R conditions, as suggested by the in situ XAFS and in situ Raman spectroscopic signals (Figures 4D and 4H). This indicates the strongly attached molecular catalysts can be alternative stabilizers to avoid significant reconstruction of Cu‐based electrocatalysts to those reported organic or inorganic modifiers [41, 42, 43]. Albeit with the slightly different ECO_2_R conditions between the real electrochemical evaluations and the in situ spectroscopic experiments, nearly 50% of Cu(I) species was retained, with approximately half of it converted to Cu(0) (Figures 4D and 4H); this strongly favors the formation of Cu(I)Cu(0) pairs as the key active sites for C‐C coupling and thus the C_2+_ production [64, 65, 66, 67, 68, 69, 70, 71, 72, 73, 74, 75]. Additionally, the in situ XAFS data also indicates the retention of molecular nature and oxidation states of the immobilized MPc complexes during electrocatalysis (Figure 4), which suggests the rapid recovery of the reactive low‐valent Ni species during CO_2_‐CO conversion and, more importantly, their stable configurations for well‐eliciting their structure‐activity relationship. Based on our in situ XAS results and TEM after electrolysis, the MPcs were relatively stable and did not decompose to metal nanoparticles. However, the results indicate the reconstruction of Cu_2_O nanocubes into metallic Cu nanostructures with hollow features, indicating the instability of Cu‐based electrocatalysts during long‐term electrolysis.

Further attention was paid to the vibrational signals of the adsorbed intermediates during ECO_2_R (Figure 5) with DFT‐based assignments. The formation of *C_1_ (mainly *CO and *CHO) species could be noticed among all these Cu‐based molecular hybrid electrocatalysts, and the *CO‐forming kinetics are highly relevant to the CO‐evolving ability of the immobilized MPc complexes, where the two thiophene‐tethered systems have shown much stronger *CO signals than those of **NiPc@Cu_2_O** during ECO_2_R. Unexpectedly, the key *C_2_ intermediate, specifically *OCCO, was only detected in the **CoPc‐OTh@Cu_2_O** system (Figures 5C and 5F), indicative of the impeded conversion of this species and thereby the less improved C_2+_ production.

With the above in situ spectroscopic observations, clear electronic and energetic landscapes for uncovering the structure‐activity relationship have been provided by systematic DFT calculations. Two critical reaction steps, CO transfer and *OCCO‐*OCCOH conversion, were taken into consideration. Both steps are thermodynamically downhill and thus governed by their kinetic activation energy barriers. Consistent with its highest activity toward CO_2_‐C_2+_ conversion, **NiPc‐OTh@Cu_2_O** displays the lowest barriers in both steps (Figures 6 and 7). During the CO‐transfer reaction, the optimal orientation of the CO molecule in the transition‐state structure and the most overlapped density of state in the corresponding Cu‐*CO intermediates contribute to the most favorable CO transfer kinetics in the **NiPc‐OTh@Cu_2_O** system (Figure 6 and Figure S45). On the other hand, during the latter *OCCO‐*OCCOH conversion, the **NiPc‐OTh@Cu_2_O** system also possesses the optimal metal‐H bond strength to significantly reduce the kinetic barrier (Figure 7 and Figure S47). In contrast, the H adsorption at the surface Cu atoms rather than the Co center within **CoPc‐OTh@Cu_2_O** causes the more kinetically difficult *OCCO hydrogenation and thereby the detectable accumulation of its *OCCO vibrational signals. However, it should be noted that the chargeless models used in the above DFT efforts fail to correlate with the positive effects from the abundant formation of Cu(I)Cu(0) diatomic active sites, which nonetheless should contribute to the ideal microenvironment for ECO_2_R (Figures 4D and 4H).

Consequently, the 2‐(3‐thienyl)ethoxy anchors and the nickel‐centered macrocycle within the **NiPc‐OTh@Cu_2_O** molecular hybrid synergistically achieve the highly retained Cu(I) species and the electronically optimal Ni center; this optimal Cu‐S‐Ni‐involved microenvironment enables the ideal energetics when reacting with *CO, *H and *C_2_ adsorbates, highlighting the crucial role of metal‐ligand cooperativity for boosting ECO_2_R to C_2+_ products. We believe our proofs of concept should demonstrate the delicate design of Cu‐based molecular hybrids toward enhancing their structural stability and CO_2_‐C_2+_ conversion efficiency, which ultimately fills the gap between mechanistic insights and structure‐activity relationship for Cu‐catalyzed C‐C coupling processes.

## Conflicts of Interest

The authors declare no conflicts of interest.

## Supporting information




**Supporting File**: exp270231‐sup‐0001‐SuppMat.docx.

## Data Availability

All necessary data for the evaluation of the conclusions drawn in this paper are available in the paper and/or the Supporting Information.
