# Supplementary material for: Unveiling Structure‐Activity Relationship With Structurally Defined and Stabilized Cu‐Based Molecular Hybrid Electrocatalysts in CO2 Electroreduction
Source: Exploration (Beijing). 2026 Oct 13;6(5):70231. doi: 10.1002/exp2.70231 (PMC13661810; doi:10.1002/exp2.70231)
Supplement: Supplementary file 1 [file EXP2-6-70231-s001.docx]

**Supporting Information**

**1. Experimental methods**

**1.1 Materials**

Carbon fiber paper (CFP; Sigracet 39 AA, 280 μm thickness, Fuel Cell Store), **NiPc** (85% dye content), multi-walled CNTs (>98% carbon basis, O.D. × L 6-13 nm × 2.5-20 μm), KHCO_3_ (≥ 99.95%), L-ascorbic acid, and polytetrafluoroethylene (PTFE) were purchased from Sigma-Aldrich. Sodium hydroxide, copper sulfate, nickel acetate tetrahydrate, 1-pentanol, 1,8-diazabicyclo [4.5.0] undec-7-ene (DBU), 4-nitrophthalonitrile, and 3-thiophene ethanol were obtained from Aladdin. All chemicals are commercially available and used without further purification.

**1.2 Instrumentation**

UV-vis spectra were collected on a UV-vis spectrometer (UV-3600, Shimadzu). Electrochemical measurements were carried out using an electrochemical workstation (CHI 660D). Gas chromatographic analysis was conducted on a FULI GC9790plus gas chromatography equipped with a thermal conductivity detector (TCD) and a flame ionization detector (FID) detector. The liquid phase of the reaction system was analyzed by ^1^H NMR.^1^ The metal contents in the composite samples were determined via inductively coupled plasma mass spectrometry (ICP-MS, iCAP Qc). Powder X-ray diffraction (PXRD) data were collected on an X-ray diffractometer (Bruker D8 Advance). X-ray photoelectron spectroscopy (XPS) data were collected on an Thermo ESCALAB 250XI instrument. Transmission electron microscopy (TEM) was operated on an electron microscope (JEOL F200). All experiments were operated at room temperature (24~25 °C) unless otherwise stated.

**1.3 Synthesis of** **NiPc-OTh**

4-[2-(3-Thienyl)ethoxy]phthalonitrile was synthesized by the reported procedure for 4-[2-(2-Thienyl)ethoxy]phthalonitrile.^2^ 4-nitrophthalonitrile (0.4 g, 2.3 mmol), 2-(3-thienyl)ethanol (1.5 mL, 10 mmol) and dry K_2_CO_3_ (0.8 g, 5.8 mmol) were mixed in 20 mL dry DMSO and stirred under argon atmosphere at room temperature for 2 days. Another portion of dry K_2_CO_3_ (0.8 g, 5.8 mmol) was added after the 2 days and the mixture was stirred under argon for another 5 days. The product was precipitated upon addition of 0.1 M HCl to near neutral pH, giving a greenish sticky residue which was rinsed with distilled water and cool acetone. After drying under vacuum, the crude product was directly used in the next step.

Later, following a general procedure to synthesize nickel phthalocyanine,^3^ [2-(3-thienyl)ethoxy]phthalonitrile (0.30 g. 1.17 mmol), five drops of 1,8-diazabicyclo[4.5.0]undec-7-ene (DBU) and nickel acetate hydrate (0.29 g, 1.17 mmol) were mixed in 10 mL of pentanol under refluxed for 1 day. The addition of hexane resulted in precipitaion which was isolated by filtration, followed by washing with hot ethyl acetate, acetone. After drying under vacuum, the crude product was purified with column chromatography by using silica gel and CHCl_3_ / CH_3_OH (5 v%) / THF (5%) eluents. Yield: 21% (88 mg) based on the phthalonitrile reagent. UV-vis (THF): *λ*_max_ [log(*ε* / M^–1^ cm^–1^)] = 279 (4.97), 318 (4.67), 382 (4.47), 609 (4.59), 673 (5.09). HR-MS *(MALDI-TOF)*: [C_56_H_40_N_8_NiO_4_S_4_] (measured: 1074.1406; simulated: 1074.141).

**1.4 Synthesis of CoPc-OTh**

Similarly, following a general procedure to synthesize cobalt phthalocyanine,^3^ [2-(3-thienyl)ethoxy]phthalonitrile (0.30 g. 1.17 mmol), five drops of 1,8-diazabicyclo[4.5.0]undec-7-ene (DBU) and cobalt acetate hydrate (0.29 g, 1.17 mmol) were mixed in 10 mL of pentanol under refluxed for 1 day. The addition of hexane resulted in precipitaion which was isolated by filtration, followed by washing with hot ethyl acetate, acetone. After drying under vacuum, the crude product was purified with column chromatography by using silica gel and CHCl_3_ / CH_3_OH (5 v%) / THF (5%) eluents. Yield: 23% (96 mg) based on the phthalonitrile reagent. UV-vis (THF): *λ*_max_ (*ε* / M^–1^ cm^–1^) = 282 (2.81), 3 (3.93), 666 (4.19). HR-MS *(MALDI-TOF)*: [C_56_H_40_N_8_CoO_4_S_4_] (measured: 1075.168; simulated: 1075.139).

**1.4 Cyclic voltammetry for homogeneous systems**

Cyclic voltammetry was performed in a 15 mL gas-tight three-neck flask with a three-electrode system, where a 3 mm glassy carbon disc electrode, a platinum wire auxiliary electrode and a AgNO_3_ (0.01 M in CH_3_CN)/Ag wire reference electrode were placed in a 0.1 M ^n^Bu_4_NPF_6_ CH_3_CN solution containing catalyst (2.0 mL). Prior to experiments, the working electrode was polished in turn with 0.3 and 0.05 *μ*m Al_2_O_3_ slurry for 3 min to obtain a mirror surface, then sonicated in water for ~60 seconds to remove debris, and finally rinsed with water. The solution was bubbled with Ar/CO_2_ for 15 min prior to each experiment. All potentials in the organic phases were referenced against ferrocenium/ferrocene (Fc^+^/Fc) as an internal standard.

**1.5 Immobilization of molecular catalysts on CNTs^4^**

40 mg CNT were pre-dispersed in 20 mL DMF by sonication for 30 min, then 3.40 μmol of **NiPc-OTh**, **NiPc** or **CoPc-OTh** dissolved in 20 mL DMF was added to the CNT suspension and stirred at room temperature for 1 h. The final materials were obtained by centrifugation and washed with DMF until the supernatant was colorless, followed by rinsing with water and final lyophilization.

**1.6 Electrochemical tests in H-cell**

The two gas-tight chambers of H-cell was separated by a Nafion 117 membrane. One chamber consists of a CFP with the prepared catalysts as the working electrode and an Ag/AgCl with saturated KCl solution as reference electrode. The other one chamber contains a platinum plate as a counter electrode. To prepare the ink for drop-casting on the working electrode, 1 mg of catalyst was dispersed into the mixture of 950 μL isopropanol and 50 μL Nafion (5 wt%) with sonication for 1 h. The ink then was dropped onto the CC with a loading of 0.1 mg cm^-2^. For ECO_2_R, 0.5 M KHCO_3_ electrolyte pre-saturated with CO_2_ (99.99%) was used as electrolyte. The CO_2_ was no longer flowed or bubbled into system during bulk electrolysis. The current densities were calculated based on the geometrical area of the electrode. In the test of H-cell, all record potentials converted into vs. RHE with 85% *iR* compensation.^5^ The generated gases in the cathodic headspace were analyzed by a gas chromatography, and the possible products in the solution were analyzed by ^1^H NMR. The Faradaic efficiency (FE) of gas product was calculated via Equation S1.

$\begin{aligned} \mathrm{FE}\left( \% \right)= \frac{Q_{\mathrm{product}}}{Q_{\mathrm{total}}}\times100\%\boldsymbol{\#}\left( S1 \right) \end{aligned}$

The measured potential was converted into the reversible hydrogen electrode (RHE) scale using the Nernst equation (Equation S2):

$$\begin{aligned} E_{\mathrm{RHE}}= E_{Ag/AgCl} + 0.197 V + 0.0591 \times pH\boldsymbol{\#}\left( S2 \right) \end{aligned}$$

The pH value of 0.5 M KHCO_3_ under argon was measured as 8.28 and that under 1 atm CO_2_ was 7.04. The pH value of 0.1 M KHCO_3_ under argon was measured as 8.20 and that under 1 atm CO_2_ was 6.81.

The partial current density is calculated using the following Equation S3:

$$\begin{aligned} j_{\mathrm{partial}}=j_{\mathrm{total}}\times\frac{\mathrm{FE}}{100}\boldsymbol{\#}\left( S3 \right) \end{aligned}$$

Where *j*_total_ is the total current density (mA·cm^-2^); FE is the Faradaic efficiency (%) for the target product.

**1.7 Electrochemical tests in flow-cell**

A three-compartment electrochemical cell (purchased from Gauss Union Co. Ltd.) consisting of a gas chamber, cathode chamber, and anode chamber was used for the three-electrode gas diffusion electrode (GDE) experiment. The electrolysis window was set to 0.5 × 2 cm^2^, yielding an active area of 1.0 cm^2^. For GDE preparation, carbon paper was first pretreated by drop-casting 60 μL of 1.0 wt% PTFE solution (diluted from a 60 wt% stock solution) onto a 1 × 3 cm^2^ gas diffusion layer (GDL, YLS-30T), followed by heating at 330 °C under an argon atmosphere for 1 hour to remove surfactants. Subsequently, 50 μL of 5.0 wt% Nafion solution, 1.6 mL deionized water, and 400 μL of 1.0 wt% PTFE solution were added to 5 mL of pre-sonicated catalyst ink, and the mixture was ultrasonicated for 1 hour. The catalyst ink was then drop-cast onto the pretreated carbon paper with a catalyst loading of 0.5 mg cm^-2^. Electrochemical measurements were conducted using a platinum foil as the counter electrode and an Ag/AgCl electrode (inserted into the cathode chamber) as the reference electrode. The cathode and anode chambers were separated by an anion exchange membrane (Selemion DSV), and 1 M KOH was used as the electrolyte at a flow rate of 15 mL/min, while CO_2_ was supplied at 30 mL/min. Current density was calculated based on the geometric area of the electrolysis window. In flow-cell testing, all recorded potentials were converted to vs. RHE with 85% iR compensation. Gas-phase products were analyzed using GC, while possible liquid-phase products were identified via ¹H NMR spectroscopy. The FE of gas-phase products was calculated using Equation S1. The cathode energy efficiency (CAEE) was calculated via Equation S4.

$$\begin{aligned} CAEE=\sum\frac{{FE}_{C_{2+}}\times E_{C_{2+}}}{1.23-E_{{CO}_{2}RR}}\boldsymbol{\#}\left( S4 \right) \end{aligned}$$

Where：

$$\begin{aligned} E_{C_{2+}}=\frac{\Delta G^{o}}{-nF}\boldsymbol{\#}\left( S5 \right) \end{aligned}$$

The full cell energy efficiency is calculated as follows S6:

$$\begin{aligned} {EE}_{cell}=\sum\frac{{FE}_{C_{2+}}\times E_{C_{2+}}}{E_{cell}}\boldsymbol{\#}\left( S6 \right) \end{aligned}$$

where ${FE}_{C_{2+}}$is the Faradaic Efficiency of C_2+_ products (ethylene, ethanol, acetate, n-propyl alcohol); $E_{C_{2+}}$is the thermodynamic cell potential for C_2+_ products (i.e.$E_{C_{2+}}$=1.17 V for ethylene); 1.23 V is the thermodynamic potential for water oxidation in the anode side; and $E_{{CO}_{2}RR}$ is the applied potential vs. RHE after iR compensation; $\Delta G^{o}$ represents the change in Gibbs free energy of the reaction, *n* represents the number of electrons required to produce one molecule of C_2+_ products, *F* is Faraday’s constant, which has a value of 96,485 C mol^−1^, $E_{cell}$ is the full-cell voltage without ohmic loss correction evaluated in the slim flow cell.

**1.8 Quantifications of products from ECO_2_R**

The gaseous products were quantitatively analyzed by a gas chromatograph (FULI INSTRUMENTS GC9790Plus) equipped with a TCD and FID detector. The TCD detector allows to analyze H_2_ and CO while the FID detector analyzes CH_4_ and C_2_H_4_. The integrals of these gaseous products were calibrated by injecting a standard gas mixture with 1% of each component in argon.

The liquid products were quantitatively analyzed by ^1^H NMR via a water-suppression technique. The following protocol was modified from a reported method^1^ by using a different volume of D_2_O. The ^1^H NMR sample was prepared by combining 450 μL of the electrolyte and 50 μL of D_2_O solution which contains 5.0 mM DMSO. Then the amounts of HCOO^-^ (8.44, 1H, s); C_2_H_5_OH (1.17, 3H, t; 3.63, 2H, q), CH_3_COO^-^ (1.9, 1H, s); and isopropanol (0.89, 3H, t; 1.54, 2H, m; 3.56, 2H, t) were calculated by integrating their proton signals and referencing the integrals of DMSO (0.5 mM).

**1.9 Synthesis of Cu_2_O nanocubes****^6^**

The whole system was kept in a 35 °C water bath with a stir bar at 600 rpm along the synthesis. We noted that the solutions subsequently added were all freshly prepared and not pre-heated to 35 °C. 2 mL of 0.1 M CuSO_4_ solution were diluted in 180 mL H_2_O in a 500 mL conical flask. 8.5 mL of 1.0 M NaOH solution were first added, followed by instant addition of 10 mL of 0.2 M ascorbic acid solution to induce orange precipitation. Then the mixture was stirred for 12 min for particle growth. The obtained nanoparticles were washed three times with a CH_3_OH:H_2_O mixture (1:1) and two times with CH_3_OH, collected by centrifugation at 7500 rpm for 5 min, and then stored in 10 mL N_2_-saturated THF to reach a concentration of 1.0 mg mL^-1^.

**1.10 Preparation of Cu-based molecular electrodes**

Certain volume (e.g., 100 μL corresponds to 10 nmol cm^-2^ loading on CFP, assuming that they are fully loaded) of 1.0 mM THF solution of **NiPc-OTh**, **NiPc** or **CoPc-OTh** was added into 1.0 mL suspension of Cu_2_O nanocubes, followed by sonication for 1 hour, giving rise to a catalyst ink. 5.0 v% of this ink (e.g., 55 μL in the case of 10 nmol cm^-2^ loading) was dropped onto a piece of CFP (0.5 cm × 2.0 cm, coverage area: ca. 0.5 cm × 1.0 cm), followed by drying under N_2_ stream for subsequent electrolysis.

**1.11 Computational detail**

**1.11.1 DFT calculation of adsorption energies (Δ*E*_ads_)**

DFT calculations were conducted through the Vienna ab initio Simulation Package (VASP) with the projector augment wave method^7,8^. Generalized gradient approximation of the Perdew-Burke-Ernzerhof (PBE) functional was used as the exchange-correlation functional^9^. The Brillouin zone was sampled with gamma points for surface calculation^10^. The cutoff energy was set as 500 eV, and structure relaxation was performed until the convergence criteria of energy and force reached 1 × 10^–5^ eV and 0.02 eV Å^–1^, respectively. For surface calculations, the **Cu_2_O(111)** surface was modeled with a periodic slab approach, and a **15 Å vacuum layer** was introduced to prevent spurious interactions between periodic images. The van der Waals (vdW) interaction was amended by the zero damping DFT-D3 method of Grimme^11^.

The adsorption energy (ΔE_ads_) of adsorbate adsorption on surface is defined as

ΔE_ads_ = E_total_ − E_slab_ − E_adsorbent_

where E_total_ and E_slab_ are the total energy of surface systems with and without adsorbate, respectively, E_adsorbent_ is the energy of an isolated adsorbate. According to this definition, negative adsorption energy suggests that the adsorption process is exothermic and the adsorption system is thermodynamically stable. Contrarily, a positive value corresponds to an endothermic and unstable adsorption.

**1.11.2 DFT calculation of ECO_2_R mechanism**

DFT calculations are performed via the Vienna ab initio simulation package (VASP)^12,13^. The initial structure was obtained through the Material Project database^14^. Ion-electron interactions are described by projection-augmented waves (PAWs). The exchange-related functional is represented by the Perdew-Burke-Ernzerhof (PBE) functional under the Generalized Gradient Approximation (GGA)^9^.

The kinetic energy cut-off for geometrically optimized was set to 450 eV, and a (3 × 3 × 1) k-point was used for the sampling of the Brillouin zone. The height of vacuum layer was set to 15 Å along the z-direction to avoid the interaction caused by periodic mirroring. Total structure was optimized on this basis until the energy and force reached the convergence thresholds of 10-5 eV and 0.03 eV/Å, respectively. The dispersion correction of DFT-D3 was introduced into the whole calculation process. The VSPKIT package and VESTA software were used to assist in the analysis of the calculation results^10^. The adsorbtion energy (${\Delta E}_{ads}$) calculated follow the formula:

$${\Delta E}_{ads}=E_{total}-E_{slab}-E_{adsorbent}$$

where $E_{total}$ and $E_{slab}$ are the total energy of surface systems with and without adsorbate, respectively, $E_{adsorbent}$ is the energy of an isolated adsorbate.

**1.12 In-situ Electrochemical Raman Spectroscopy Measurement**

In-situ electrochemical Raman spectroscopy measurements were performed using HORIBA LabRAM Odyssey with an excitation wavelength of 532 nm. The electrochemical test was conducted in 0.1 M KHCO₃ CO_2_-saturated solution as the electrolyte, applying potentials from 0 to -2 V vs Ag/AgCl (with 0.1 V interval). After 10s electrolysis at each potential, Raman spectra were recorded to monitor the in-situ electrochemical Raman spectra at the working electrode interface. The working electrode was prepared by drop-casting a molecular-copper-based catalyst ink onto GDL carbon paper with a catalyst loading of 5 mg cm^-2^. A Pt mesh was used as the counter electrode, and an Ag/AgCl electrode served as the reference electrode. The entire electrode system was assembled into an in-situ electrochemical Raman reaction cell. All potentials reported in the main text given vs. RHE without iR compensation.

**1.13 In-situ Electrochemical FTIR Spectroscopy Measurement**

in-situ electrochemical FTIR spectroscopy measurements were conducted using a Nicolet™ iS50 FTIR infrared spectrometer, equipped with a liquid nitrogen-cooled MCT (Mercury-Cadmium-Telluride) detector. The electrochemical test was performed in 0.1 M KHCO₃ CO_2_-saturated solution as the electrolyte, applying potentials from 0 to -2 V vs Ag/AgCl (with 0.1 V interval). After 10s electrolysis at each potential, FTIR spectra were recorded to monitor the in-situ electrochemical infrared spectra at the working electrode interface. The working electrode was prepared by drop-casting a molecular-copper-based catalyst ink onto GDL carbon paper with a catalyst loading of 5 mg cm^-2^. A Pt mesh was used as the counter electrode, and an Ag/AgCl electrode served as the reference electrode. The entire electrode system was assembled into an in-situ electrochemical infrared reaction cell. All potentials reported in the main text given vs. RHE without iR compensation.

**1.14 In-situ XAFS Measurement**

The XAFS spectra at the Ni and Cu K-edge in fluorescence mode were collected at the Energy Materials Beamline-hard branch (31124.02.SSRF.BL20U1) and BL17B beamline at SSRF. The electron beam energy was 3.5 GeV and the stored current was 230 mA (top-up). The XAFS data were collected using a fixed-exit double-crystal Si(111) monochromator. The fluorescence signals of Ni and Cu were collected using a Lytle detector. The energy was calibrated using Ni foil and Cu foil.

The in-situ XAFS spectra were collected with a home-made cell under the same conditions as used for the electrochemical measurements. For the in-situ measurements, 0.1 M aqueous KHCO_3_ was used as the electrolyte, and Ag/AgCl and Pt were used as the reference and counter electrodes, respectively. A 1 cm × 1 cm piece of the hydrophobic carbon paper with a catalyst loading of 2 mg cm^−2^ was used as the working electrode. During the measurements, each applied potential was maintained for at least 30 min before the collection of spectra to ensure the stability of the current. All potentials reported in the main text given vs. RHE without iR compensation.

The raw data was analyzed using the IFEFFIT software package according to the standard data analysis procedures. The spectra were calibrated, averaged, pre-edge background subtracted, and post-edge normalized using the Athena program in the IFEFFIT software package. The Fourier transformation of the *k*^3^-weighted EXAFS oscillations, *k*^3^·χ(k), from k space to R space was performed to obtain a radial distribution function. The data fitting was performed using the Artemis program in the IFEFFIT software package.

**Supporting Figures and Tables**

**Figure S1.** UV-vis absorption spectra of 5.0 μM **NiPc-OTh** in THF.

**Figure S2.** UV-vis absorption spectra of 1.0 μM **CoPc-OTh** in THF.

**Figure S3.** MALDI-TOF accurate mass spectrum of **NiPc-OTh.**

**Figure S4.** MALDI-TOF accurate mass spectrum of **CoPc-OTh.**

**Figure S5.** CVs of 1.0 mM **NiPc-OTh** in DMF containing 0.1 M TBAPF_6_ under Ar or CO_2_ at a scan rate of 100 mV s^−1^.

**Figure S6.** CVs of 1.0 mM **CoPc-OTh** in DMF containing 0.1 M TBAPF_6_ under Ar or CO_2_ at a scan rate of 100 mV s^−1^.

**Figure S7.** EDS elemental mapping on **NiPc-OTh@CNT**. Color: C (orange), Ni (green), O (red), S (yellow), N (blue).

**Figure S8.** EDS elemental mapping on **NiPc@CNT**. Color: C (orange), Ni (green), O (red), N (blue).

**Figure S9.** EDS elemental mapping on **NiPc@CNT**. Color: C (orange), Ni (green), O (red), N (blue).

**Figure S10.** Current density traces from chronoamperometry at varying potentials in 0.5 M KHCO_3_ solutions with (A) **NiPc @CNT**, (B) **NiPc-OTh@CNT** or (C) **CoPc-OTh@CNT**.

**Figure S11.** PXRD patterns of Cu_2_O nanocubes. A standard XRD pattern of Cu_2_O is also shown.

**Figure S12.** (A) TEM and (B) HRTEM images of Cu_2_O. Inset in (B) shows the FFT mode.

**Figure S13.** Photo of suspensions (left to right) of **NiPc@Cu_2_O**, bare Cu_2_O and **NiPc-OTh@Cu_2_O**.

**Figure S14.** UV-vis absorption spectra of 100 μL 1.0 mM THF solution of **NiPc-OTh** (red) or **NiPc** (blue) added into 1.0 mL THF (bright red/blue) or the first centrifuge supernatant after immobilization by 1.0 mL THF suspension of Cu_2_O nanocubes (pale red/blue), showing the rough adsorption ratio of the nickel complexes on Cu-based surface.

**Figure S15.** UV-vis absorption spectra of 100 μL 1.0 mM THF solution of **CoPc-OTh** added into 1.0 mL THF (orange) or the first centrifuge supernatant after immobilization by 1.0 mL THF suspension of Cu_2_O nanocubes (pale orange), showing the rough adsorption ratio of **CoPc-OTh** on Cu_2_O surface.

**Figure S16.** EDS elemental mapping on as-prepared **NiPc-OTh@Cu_2_O**. Color: Cu (orange), Ni (green), O (red), S (violet), N (blue).

**Figure S17.** EDS elemental mapping on as-prepared **NiPc@Cu_2_O**. Color: Cu (green), N (blue), Ni (light blue), O (pink).

**Figure S18.** EDS elemental mapping on as-prepared **CoPc-OTh@Cu_2_O**. Color: Cu (orange), Co (red), O (light blue), S (violet), N (blue).

**Figure S19.** XPS spectra of Cu 2p for (A) bare Cu_2_O, (B) **NiPc@Cu_2_O**, (C) **NiPc-OTh@Cu_2_O**, (D) **CoPc-OTh@Cu_2_O**.

**Figure S20.** XPS spectra of Ni 2p for **NiPc-OTh** and **NiPc-OTh@Cu_2_O**.

**Figure S21.** XPS spectra of S 2p for **NiPc-OTh** and **NiPc-OTh@Cu_2_O**.

**Figure S22.** XPS spectra of Co 2p for **CoPc-OTh** and **CoPc-OTh@Cu_2_O**.

**Figure S23.** XPS spectra of S 2p for **CoPc-OTh** and **CoPc-OTh@Cu_2_O**.

**Figure S24.** XPS spectra of Ni 2p for **NiPc** and **NiPc@Cu_2_O**.

**Table S1.** Binding energy values (eV) in Cu 2p XPS spectra. The observed Cu(II) signal likely originates from the surface oxidation of Cu_2_O when exposed to atmospheric conditions.

|  | Cu(I) | Cu(II) | Cu(II) satellite |
| --- | --- | --- | --- |
| **Cu_2_O** | 932.80 | 934.87 | 942.47 |
| **NiPc@Cu_2_O** | 932.85 | 934.93 | 942.41 |
| **NiPc-OTh@Cu_2_O** | 932.89 | 934.63 | 942.6 |
| **CoPc@Cu_2_O** | 932.79 |  |  |
| **CoPc-OTh@Cu_2_O** | 932.60 |  |  |

**Table S2.** Binding energy values (eV) in Ni 2p XPS spectra.

|  | Ni 2p_1/2_ | Ni 2p3_/2_ |
| --- | --- | --- |
| **NiPc** | 873.00 | 855.81 |
| **NiPc@Cu_2_O** | 872.92 | 855.60 |
| **NiPc-OTh** | 872.94 | 855.60 |
| **NiPc-OTh@Cu_2_O** | 872.23 | 855.03 |

**Table S3.** Binding energy values (eV) in S 2p XPS spectra.

|  | S 2p_1/2_ | S 2p3_/2_ |
| --- | --- | --- |
| **NiPc-OTh** | 165.25 | 164.14 |
| **NiPc-OTh@Cu_2_O** | 165.89 | 164.74 |
| **CoPc-OTh** | 165.42 | 164.23 |
| **CoPc-OTh@Cu_2_O** | 165.68 | 164.47 |

**Table S4.** Binding energy values (eV) in Co 2p XPS spectra.

|  | Co 2p_1/2_ | Co 2p3_/2_ |
| --- | --- | --- |
| **CoPc-OTh** | 795.76 | 780.33 |
| **CoPc-OTh@Cu_2_O** | 796.04 | 780.55 |

**Table S5.** The calculated adsorption energy(ΔE_ads_) of different systems and the calculated energy of different substances in different systems.

| System | E(Cu_2_O (111)) | E(**NiPc**) | E(**NiPc@Cu_2_O**) | **ΔE_ads_** |
| --- | --- | --- | --- | --- |
| **NiPc@Cu_2_O** | -795.465804 | -420.761876 | -1219.358458 | **-3.13078** |
| System | E(Cu_2_O (111)) | E(**NiPc-OTh**) | E(**NiPc-OTh@Cu_2_O**) | **ΔE_ads_** |
| **NiPc-OTh@Cu_2_O** | -795.465804 | -767.200939 | -1569.875605 | **-7.20886** |
| System | E(Cu_2_O (111)) | E(**CoPc-OTh**) | E(**CoPc-OTh@Cu_2_O**) | **ΔE_ads_** |
| **CoPc-OTh@Cu_2_O** | -795.465804 | -767.909098 | -1569.664268 | **-6.28937** |

**Figure S25.** Potential-dependent performances in ECO_2_R with bars indicating FEs for Cu_2_O with varying **NiPc-OTh** loadings in a gas-tight, H-cell set-up. To optimize the performance, the loading of **NiPc-OTh** on Cu_2_O was varied, where a volcano trend was observed for the FE(C_2+_) versus the Ni loading, giving an optimal loading of 10 nmol cm^-2^. The initial activity improvement along with increasing loading is achieved by appending more active CO-evolving sites. However, the presence of excess **NiPc-OTh** leads to severe aggregations of the Cu_2_O nanocubes, which could not be evenly dispersed on CFPs at loadings over 16 nmol cm^-2^, presumably causing ineffective mass transport and high electrical resistance of the corresponding molecular hybrids.

**Table S6**. The ECO_2_RR performance of different catalysts at different cathode potentials.

| Potential (V vs. RHE） | Catalysts | *j* (mA cm^-2^) | FE (%) | | | | | | | | |
| --- | --- | --- | --- | --- | --- | --- | --- | --- | --- | --- | --- |
|  |  |  | H_2_ | CO | CH_4_ | C_2_H_4_ | Formic acid | Ethanol | Acetic acid | 1-propanol | Total C_2+_ |
| -0.96 | **Cu_2_O** | 7.30±1.32 | 34.06±3.02 | 0.00±0.00 | 12.32±0.50 | 18.19±1.50 | 2.58±0.08 | 6.43±2.45 | 0.53±0.09 | 5.62±1.03 | 30.77±1.66 |
|  | **NiPc@Cu_2_O** | 5.20±0.06 | 54.34±2.09 | 5.66±3.86 | 2.69±0.20 | 15.61±0.56 | 4.01±0.24 | 3.27±1.62 | 0.00±0.00 | 0.00±0.00 | 18.88±2.18 |
|  | **NiPc-OTh@Cu_2_O** | 6.20±0.18 | 27.59±2.69 | 4.81±1.56 | 3.06±0.09 | 34.58±1.70 | 3.62±0.06 | 8.79±1.64 | 0.40±0.35 | 9.36±0.27 | 53.14±3.04 |
|  | **NiPc-OTh@Cu_2_O** (-0.90 V)* | 271.74±14.13 | 11.67±6.56 | 20.89±5.38 | 1.54±0.25 | 36.63±5.49 | 3.85±0.65 | 13.74±6.51 | 1.61±0.47 | 5.51±0.84 | 57.50±4.66 |
|  | **CoPc-OTh@Cu_2_O** | 7.60±1.11 | 37.57±5.85 | 17.60±5.83 | 1.91±0.71 | 19.26±0.17 | 2.66±0.42 | 0.00±0.00 | 0.00±0.00 | 0.00±0.00 | 19.26±0.77 |
| -1.00 | **Cu_2_O** | 9.92±1.05 | 29.14±0.59 | 1.77±0.70 | 12.64±1.18 | 22.04±0.77 | 3.05±0.30 | 7.83±0.78 | 0.39±0.04 | 7.83±0.78 | 38.09±2.20 |
|  | **NiPc@Cu_2_O** | 8.20±1.14 | 43.41±2.07 | 0.83±1.43 | 14.23±2.92 | 25.30±2.98 | 2.10±0.75 | 6.12±0.50 | 0.12±0.21 | 0.00±0.00 | 31.54±3.70 |
|  | **NiPc-OTh@Cu_2_O** | 9.40±0.51 | 19.91±5.29 | 1.99±0.36 | 6.53±0367 | 40.74±2.06 | 2.58±0.31 | 9.48±0.98 | 0.69±0.26 | 8.96±1.61 | 59.87±4.52 |
|  | **NiPc-OTh@Cu_2_O** (-0.96 V)* | 347.48±10.93 | 8.59±1.79 | 6.91±4.84 | 3.86±2.55 | 59.04±4.50 | 0.88±2.64 | 15.25±7.18 | 2.70±0.85 | 1.32±1.86 | 79.19±1.29 |
|  | **CoPc-OTh@Cu_2_O** | 9.30±0.79 | 33.30±1.74 | 6.70±3.50 | 9.60±1.66 | 31.50±2.26 | 2.81±0.07 | 3.94±1.12 | 0.56±0.30 | 3.95±3.42 | 39.95±1.77 |
| -1.04 | **Cu_2_O** | 12.30±0.61 | 32.11±4.62 | 2.52±1.05 | 18.75±3.10 | 25.79±1.97 | 2.13±0.04 | 5.33±0.53 | 0.31±0.01 | 5.16±2.11 | 36.59±4.29 |
|  | **NiPc@Cu_2_O** | 10.25±0.83 | 48.77±2.35 | 0.49±0.84 | 13.28±0.53 | 25.72±0.44 | 2.21±0.10 | 0.74±0.64 | 0.00±0.00 | 0.00±0.00 | 26.46±1.10 |
|  | **NiPc-OTh@Cu_2_O** | 10.77±0.16 | 14.64±1.88 | 1.87±0.43 | 6.56±1.45 | 49.24±2.44 | 1.97±0.45 | 9.28±1.10 | 1.07±0.37 | 8.35±0.92 | 67.95±1.38 |
|  | **NiPc-OTh@Cu_2_O** (-1.06 V)* | 361.52±12.37 | 13.00±5.66 | 14.93±8.45 | 10.66±3.49 | 46.80±8.94 | 2.38±1.32 | 6.84±3.38 | 2.81±1.16 | 2.67±1.48 | 59.11±8.70 |
|  | **CoPc-OTh@Cu_2_O** | 10.40±1.44 | 28.71±5.03 | 8.03±2.52 | 13.47±1.44 | 37.25±3.13 | 2.05±0.40 | 3.68±1.06 | 0.66±0.56 | 4.18±1.71 | 45.77±3.27 |
| -1.08 | **Cu_2_O** | 14.13±0.58 | 31.91±6.60 | 2.39±0.07 | 17.66±2.06 | 24.59±2.81 | 1.35±0.08 | 8.29±0.79 | 0.36±0.14 | 6.81±1.14 | 40.04±4.55 |
|  | **NiPc@Cu_2_O** | 11.19±0.70 | 49.74±7.40 | 1.22±0.25 | 11.28±3.66 | 25.42±4.01 | 1.25±0.76 | 2.73±1.65 | 0.64±0.26 | 1.91±0.19 | 30.69±6.10 |
|  | **NiPc-OTh@Cu_2_O** | -11.60±2.11 | 18.22±3.89 | 0.79±0.17 | 13.73±2.01 | 42.77±3.16 | 1.79±0.33 | 7.82±1.93 | 0.76±0.09 | 6.22±1.62 | 57.56±1.81 |
|  | **NiPc-OTh@Cu_2_O** (-1.12 V)* | 271.17±8.49 | 9.05±5.35 | 18.61±3.14 | 6.02±2.72 | 46.71±4.73 | 1.85±1.26 | 9.71±5.63 | 3.31±1.58 | 2.17±0.58 | 61.90±4.17 |
|  | **CoPc-OTh@Cu_2_O** | 11.60±0.66 | 27.16±1.25 | 6.44±2.68 | 17.63±5.12 | 37.66±4.31 | 1.57±0.21 | 4.17±1.01 | 0.54±0.17 | 4.37±1.62 | 46.74±3.02 |

The asterisk (*) denotes the ECO_2_RR performance of NiPc-OTh@Cu_2_O in a GDE system, while all other data were obtained in an H-cell.

**Table S7**. Comparison of the ECO_2_R performance of recently reported non-precious metal copper based tandem electrocatalysts.

| Catalysts | Electrolyte | V vs. RHE | $\text{FE}_{\text{C}_{\text{2}}\text{H}_{\text{4}}}$/% | $j_{\text{C}_{\text{2}}\text{H}_{\text{4}}}$/mA cm^–2^ | Ref. |
| --- | --- | --- | --- | --- | --- |
| **NiPc-OTh@Cu_2_O** | 0.5 M KHCO_3_ | -1.04 | 49±2 | 4.8 | This work |
| Cu/Ni-NAC | 0.5 M KHCO_3_ | –1.4 | 50 | - | 15 |
| CuPOF-Bpda/Cu_2_O@CNT | 0.5 M KHCO_3_ | –1.1 | 53.5 | 12.8 | 16 |
| CoPc-Cucub/C | 1 M KHCO_3_ | −1.05 | 48(C_2+_) | 1.5(C_2+_) | 17 |
| CuO/Cu-MOF | 0.1 M KHCO_3_ | –1.1 | 50 | 7 | 18 |
| Cu(111)@Cu-THQ | 0.1 M KHCO_3_ | –1.2 | 44.2 | 8.2 | 19 |
| Cu-MOF-CF | 0.1 M KHCO_3_ | –1.11 | 48.6 | 8.3 | 20 |
| Fe-TCPP@Cu | 0.5 M KHCO_3_ | –1.17 | 33.42 | 12.5 | 21 |
| PTF/Cu | 0.1 M KHCO_3_ | -1.1 | 27.9 | 0.2 | 22 |
| Cucub/Fe-TBTPP | 1 M KHCO_3_ | −1.05 | 36 | - | 23 |

**Figure S26.** EDS elemental mapping on **NiPc-OTh@Cu_2_O** after electrolysis at -1.04 V vs RHE for 1 h. The sample was collected by sonication of the working electrode in ethanol for 30 min and then dispersed on a Cu grid for analysis. Color: Cu (orange), Ni (green), O (red), S (violet), N (blue).

**Figure S27.** EDS elemental mapping on **NiPc@Cu_2_O** after electrolysis at -1.04 V vs RHE for 1 h. The sample was collected by sonication of the working electrode in ethanol for 30 min and then dispersed on a Cu grid for analysis. Color: Cu (green), N (blue), Ni (light blue), O (pink). The reconstruction could be more severe than the OTh-anchored cases (Figure S26 and S28) for its larger holes in the cubes.

**Figure S28.** EDS elemental mapping on **CoPc-OTh@Cu_2_O** after electrolysis at -1.04 V vs RHE for 1 h. The sample was collected by sonication of the working electrode in ethanol for 30 min and then dispersed on a Cu grid for analysis. Color: Color: Cu (orange), Co (red), O (light blue), S (violet), N (blue).

**Figure S29.** Potential-dependent performances in ECO_2_R with bars indicating FEs for **NiPc-OTh@Cu_2_O** in a GDE reactor. The loading of metal complex was set at 10 nmol cm^-2^.

**Figure S30.** Current density traces from chronoamperometry at -0.96 V vs RHE in 1.0 M KOH solutions with **NiPc-OTh@Cu_2_O** catalysts in a flow-cell system revealed that at high current densities (>250 mA cm^-2^), catholyte flooding and carbonate precipitation occurred, where the electrolyte inundated the **NiPc-OTh@Cu_2_O** electrode. This hindered CO_2_ transport and led to a rapid decline in both activity and selectivity within 1 hour. We attribute the limited electrolysis time to cell flooding and/or other technical issues, consistent with prior observations. Indeed, liquid droplets observed on the gas diffusion layer upon disassembly corroborate this mechanism. Overcoming these technical problems for prolonged operation remains prioritized for future investigation, while this would not affect the research target of this work which focuses on bridging mechanistic insights with reaction pathway analysis.

**Figure S31.** Fourier transform of the k^3^-weighted Cu K-edge EXAFS fitting curves for **NiPc-OTh@Cu_2_O** during ECO_2_R.

**Table S8.** Ex-situ/in-situ EXAFS structural parameters for **NiPc-OTh@Cu_2_O**.

| **Sample** | **Shell** | **N** | **R (Å)** | **σ^2^ (×10^-3^Å^2^)** | **ΔE (eV)** | **R-factor** |
| --- | --- | --- | --- | --- | --- | --- |
| Cu-foil | Cu-Cu | 12 | 2.55 | — | — | — |
| CuO | Cu-O | 4 | 1.95 | — | — | — |
| Cu_2_O | Cu-O | 2 | 1.85 | — | — | — |
| dry | Cu-O | 1.3 | 1.87 | 3.0 | 7.5 | 0.36% |
|  | Cu-Cu | 6.0 | 2.98 | 9.0 | 7.5 |  |
| OCP | Cu-O | 1.3 | 1.86 | 3.8 | 7.1 | 0.74% |
|  | Cu-Cu | 4.8 | 3.01 | 9.0 | 7.1 |  |
| -0.6 V | Cu-O | 1.2 | 1.83 | 3.0 | 4.5 | 2.00% |
|  | Cu-Cu | 0.9 | 2.54 | 6.2 | 4.5 |  |
|  | Cu-Cu | 5.0 | 2.85 | 11.5 | 4.5 |  |
| -0.8 V | Cu-O | 0.8 | 1.85 | 3.9 | 9.6 | 1.30% |
|  | Cu-Cu | 3.0 | 2.54 | 9.7 | 9.6 |  |
| -1.0 V | Cu-O | 0.6 | 1.84 | 3.6 | 7.7 | 1.34% |
|  | Cu-Cu | 3.0 | 2.55 | 8.6 | 7.7 |  |
| -1.2 V | Cu-O | 0.5 | 1.82 | 3.0 | 6.3 | 0.83% |
|  | Cu-Cu | 3.9 | 2.54 | 8.9 | 6.3 |  |

*R—distance, N—coordination number, σ^2^—Debye-Waller factor.

**Figure S32.** In-situ XANES spectra of **CoPc-OTh@Cu_2_O** at Co K-edge (A) and Cu K-edge (B). (C) in-situ *k*^3^-weight Cu K-edge FT-EXAFS spectra for **CoPc-OTh@Cu_2_O** during ECO_2_R.

**Figure S33.** Fourier transform of the *k*^3^-weighted Cu K-edge EXAFS fitting curves for **CoPc-OTh@Cu_2_O** during ECO_2_R.

**Table S9.** Ex-situ/in-situ EXAFS structural parameters for **CoPc-OTh@Cu_2_O**.

| **Sample** | **Shell** | **N** | **R (Å)** | **σ^2^ (×10^-3^Å^2^)** | **ΔE (eV)** | **R-factor** |
| --- | --- | --- | --- | --- | --- | --- |
| Cu-foil | Cu-Cu | 12 | 2.55 | — | — | — |
| CuO | Cu-O | 4 | 1.95 | — | — | — |
| Cu_2_O | Cu-O | 2 | 1.85 | — | — | — |
| dry | Cu-O | 1.6 | 1.83 | 3.0 | 6.6 | 1.95% |
|  | Cu-Cu | 2.9 | 2.94 | 9.0 | 6.6 |  |
| -1.2V | Cu-O | 1.3 | 1.83 | 3.0 | 6.2 | 1.93% |

R—distance, N—coordination number, σ^2^—Debye-Waller factor.

**Figure S34.** Fourier transform of the *k*^3^-weighted Cu K-edge EXAFS fitting curves for **NiPc@Cu_2_O** during ECO_2_R.

**Table S10.** Ex situ/in-situ EXAFS structural parameters for **NiPc@Cu_2_O**.

| **Sample** | **Shell** | **N** | **R (Å)** | **σ^2^ (×10^-3^Å^2^)** | **ΔE (eV)** | **R-factor** |
| --- | --- | --- | --- | --- | --- | --- |
| Cu-foil | Cu-Cu | 12 | 2.55 | — | — | — |
| CuO | Cu-O | 4 | 1.95 | — | — | — |
| Cu_2_O | Cu-O | 2 | 1.85 | — | — | — |
| dry | Cu-O | 2.0 | 1.94 | 4.5 | 3.7 | 1.95% |
|  | Cu-Cu | 1.2 | 3.10 | 9.0 | 3.7 |  |
| OCP | Cu-O | 2.2 | 1.94 | 5.7 | 3.6 | 2.00% |
|  | Cu-Cu | 1.3 | 3.10 | 11.0 | 3.6 |  |
| -0.6 V | Cu-O | 0.7 | 1.85 | 6.6 | 4.3 | 0.58% |
|  | Cu-Cu | 4.3 | 2.54 | 6.8 | 4.3 |  |
| -0.8 V | Cu-Cu | 6.0 | 2.53 | 7.5 | 4.3 | 0.23% |
| -1.0 V | Cu-Cu | 6.8 | 2.54 | 7.5 | 4.6 | 0.14% |
| -1.2 V | Cu-Cu | 6.9 | 2.54 | 7.9 | 4.1 | 0.12% |

*R—distance, N—coordination number, σ^2^—Debye-Waller factor.

**Figure S35.** Linear combination fitting (LCF) analysis of Cu K-edge XANES spectra for **NiPc@Cu_2_O** under different applied potentials.

LCF of the Cu K-edge XANES spectra for the **NiPc@Cu_2_O** samples was performed using Cu foil (Cu(0)), Cu_2_O (Cu(I)), and CuO (Cu(II)) as reference compounds. These standards were obtained independently and exhibit representative spectral features. The fitting results show that the XANES spectra under different applied potentials can be well reproduced by linear combinations of the three references. The fitting residuals are shown as residual curves beneath each spectrum. Their low amplitude and the absence of systematic deviations indicate that the fitting quality is high, allowing reliable quantification of the relative proportions of Cu species.

Under open circuit potential (OCP), the spectrum is dominated by CuO features, suggesting the presence of surface Cu(II) species, likely due to aerobic oxidation of Cu_2_O upon air exposure. Upon applying a potential of -0.6 V, the CuO peak disappears, indicating reduction of Cu(II) species. With further negative shifts in potential, the Cu(I) content gradually decreases while the Cu(0) fraction increases significantly, implying progressive electrochemical reduction of Cu_2_O. At −1.0 V, Cu(0) becomes the dominant species, accounting for approximately 90% of the total Cu content, indicating that most of the Cu_2_O has been reduced to the metallic state, although a small fraction of Cu(I) species remains unreduced.

**Figure S36.** LCF analysis of Cu K-edge XANES spectra for **NiPc-OTh@Cu_2_O** under different applied potentials.

For the **NiPc-OTh@Cu_2_O** sample, LCF of the Cu K-edge XANES spectra was conducted using Cu foil (Cu(0)) and Cu_2_O (Cu(I)) as reference compounds, which were independently measured and exhibit representative features. The fitting results show that the experimental spectra under different applied potentials can be effectively reproduced by linear combinations of the two references. Similar to the previous sample, the fitting residuals are presented as residual curves beneath the spectra and remain small overall, further confirming the reliability of the fitting.

At OCP, the spectrum is primarily characterized by Cu(I) features, with only a minor contribution from Cu(0). As the applied potential becomes increasingly negative, the Cu(I) content gradually decreases, while the proportion of Cu(0) slightly increases. Compared to **NiPc@Cu_2_O**, the **NiPc-OTh@Cu_2_O** sample exhibits a significantly higher Cu(I) fraction under the same catalytic potentials, indicating that the introduction of the -OTh group effectively suppresses their Cu(I)/Cu(0) reduction.

**Table S11**. Assignments of Raman-active species.

| **Species** | **Raman shift (cm^-1^)** |
| --- | --- |
| Cu_2_O | 142-150, 216-223, 525-534, 615-630 |
| Cu-CO | 276-290, 354-370 |
| CO_3_^2-^ | 1020-1030 |
| Adsorbed H_2_O | 1640-1650 |
| C≡O | 1900-2000, 2000-2120 |
| COOH | 1200-1250 |
| OCCOH | 1300-1400 |

**Table S12**. Assignments of infrared-active species.

| **Species** | **Wavenumber (cm^-1^)** |
| --- | --- |
| CHO | 1420-1460, 1730-1740 |
| OCCO | 1500-1600 |
| Adsorbed H_2_O | 1630-1650 |
| C≡O | 2040-2050 |
| HCO_3_^-^ | 2160-2170 |
| CO_3_^2-^ | 2370-2380 |

**Table S13**. The Raman shift values of *COOH reported in the literature.

| Catalysts | ***COOH Raman shift (cm^-1^)** | Ref. |
| --- | --- | --- |
| PTF(Ni)/Cu | 1387 | ^24^ |
| np-Ag | 1386, 1600 | ^25^ |
| Cu | 1200-1400 | ^26^ |
| Bi-NFs | 1130 | ^27^ |
| Cu(110) | 1046 | ^28^ |
| s-PdNi/CNFs-1000 | 1064 | ^29^ |

**Table S14**. The Raman shift values of *OCCO reported in the literature.

| Catalysts | ***OCCO Raman shift (cm^-1^)** | Ref. |
| --- | --- | --- |
| Cu single-crystal | 1005 | ^30^ |
| Cu | 1450, 1550 | ^31^ |
| PTF(Ni)/Cu | 1585 | ^24^ |
| Cu(110) | 2088 | ^28^ |
| Cu | 1189 | ^32^ |

**Figure S37.** DFT-optimized Raman fingerprints of the *COOH intermediate in **CoPc-OTh@Cu_2_O**.

**Figure S38.** DFT-optimized Raman fingerprints of the *OCCO intermediate in **CoPc-OTh@Cu_2_O**.

**Figure S39.** DFT-optimized FTIR vibrational fingerprints of the *OCCO reaction intermediate in **CoPc-OTh@Cu_2_O**.

**Figure S40. The free energy diagrams for the ECO_2_R on NiPc@Cu_2_O, NiPc-OTh@Cu_2_O, and CoPc-OTh@Cu_2_O.** The results demonstrate that C-C coupling and subsequent *OCCO-to-*OCCOH conversion are thermodynamically favorable in all three catalysts.

**Figure S41.** Side view of the structures involved in the CO transfer process for **NiPc@Cu_2_O**, **NiPc-OTh@Cu_2_O**, and **CoPc-OTh@Cu_2_O**.

**Figure S42. Crystal Orbital Hamilton Population (COHP) analysis of CO binding in NiPc@Cu_2_O, NiPc-OTh@Cu_2_O, and CoPc-OTh@Cu_2_O, with bonding and antibonding states shown on the right and left, respectively.** Projected crystal orbital Hamilton population (pCOHP) analyses reveal that the electronic structures of the MPc complexes modulate the CO-binding strengths. The results show that **NiPc** exhibits a much higher Ni-C bonding strength compared to **NiPc-OTh** and **CoPc-OTh**, with the Ni 3d and C 2p orbitals forming stronger bonding interactions. Consequently, **NiPc** effectively adsorbs CO, making its desorption and subsequent transfer to the Cu_2_O surface more difficult. In contrast, in **NiPc-OTh**, the electron-donating effect of the -OTh group donates a small number of electrons to the Ni center, partially filling the anti-bonding orbitals of the Ni-C bond, thereby weakening CO adsorption. This weakened interaction facilitates the desorption and transfer of *CO. Notably, in **CoPc-OTh**, the Co-C bond exhibits even greater filling in the anti-bonding states than **NiPc**, resulting in weaker CO adsorption.

**Figure S43.** Partial density of states (PDOS) and d-band center of the metal centers in **NiPc**, **NiPc-OTh**, and **CoPc-OTh**. The calculated d-band center values are -2.01 eV for **NiPc**, -2.67 eV for **NiPc-OTh**, and -3.55 eV for **CoPc-OTh**, consistent with the COHP results. Among them, the d-band center of Ni in **NiPc** is close to the fermi level, which enhances the adsorption of CO. This strong orbital interaction enhances CO binding, thereby impeding its desorption and transfer. In contrast, benefited from the electron-donor effect of the -OTh group and the unique electronic structure of Co, the *d*-band centers of **NiPc-OTh** and **CoPc-OTh** was shifted to lower energies, ultimately reducing the CO adsorption strength, which promotes CO transfer to the Cu_2_O surface.

**Figure S44.** The partial density of states of CO in the MPc@Cu_2_O hybrid system M-*CO intermediate with oxygen on the Cu_2_O surface: (A) **NiPc-OTh@Cu_2_O**, (B) **NiPc@Cu_2_O**, (C) **CoPc-OTh@Cu_2_O**. The overlap of density of states between CO and the Cu_2_O surface was additionally calculated to qualitatively explore the energy difference of the *CO transfer configuration on the three catalysts. In **NiPc-OTh**, the oxygen on the Cu_2_O surface has the largest overlap with the p orbital of CO, which makes the energy change more continuous and the energy barrier is further reduced.

**Figure S45.** Side view of the key C_2_ intermediate models for the optimized **NiPc@Cu_2_O**, **NiPc-OTh@Cu_2_O**, and **CoPc-OTh@Cu_2_O**.

**Figure S46.** The hydrogen adsorption models and Ni-H bond lengths of the optimized (A) **NiPc@Cu_2_O** and (B) **NiPc-OTh@Cu_2_O**.

**Figure S47.** Calculated free energy of reaction pathways for different hydrogen adsorption sites of the *OCCO intermediate in **CoPc-OTh@Cu_2_O**.

**Figure S48.** Optimized pathway model for hydrogen adsorption on Co in **CoPc-OTh@Cu_2_O**. (A) *OCCO-H; (B) TS2 (pale green path in Figure S45).

**References**

1	Chatterjee, T., Boutin, E. & Robert, M. Manifesto for the routine use of NMR for the liquid product analysis of aqueous CO_2_ reduction: from comprehensive chemical shift data to formaldehyde quantification in water. *Dalton. Trans.* **49**, 4257-4265 (2020).

2	Obirai, J. & Nyokong, T. Synthesis, electrochemical and electrocatalytic behaviour of thiophene-appended cobalt, manganese and zinc phthalocyanine complexes. *Electrochim. Acta* **50**, 5427-5434 (2005).

3	Aktas Kamiloglu, A., Saka, E. T., Acar, I. & Tekintas, K. Synthesis, characterization, and photocatalytic activity of Co(II) and Cu(II) phthalocyanines linked with thiophene–Schiff base substituents for 4-nitrophenol oxidation. *J. Coord. Chem.* **72**, 2778-2790 (2019).

4	Huang, Y. *et al.* Impaired conjugation boosts CO_2_ electroreduction by Ni(II) macrocyclic catalysts immobilized on carbon nanotubes. *J. Mater. Chem. A* **11**, 2969-2978 (2023).

5	Zheng, W. iR Compensation for Electrocatalysis Studies: Considerations and Recommendations. *ACS Energy Lett.* **8**, 1952-1958 (2023).

6	Ke, W. H., Hsia, C. F., Chen, Y. J. & Huang, M. H. Synthesis of Ultrasmall Cu_2_O Nanocubes and Octahedra with Tunable Sizes for Facet-Dependent Optical Property Examination. *Small* **12**, 3530-3534 (2016).

7	Kresse, G. From ultrasoft pseudopotentials to the projector augmented-wave method. *Phys. Rev. B Condens. Matter* **59**, 1758-1775 (1999).

8	Blöchl, P. E. Projector augmented-wave method. *Phys. Rev. B* **50**, 17953-17979 (1994).

9	Perdew, J. P., Burke, K. & Ernzerhof, M. Generalized Gradient Approximation Made Simple. *Phys. Rev. Lett.* **77**, 3865-3868 (1996).

10	Wang, V., Xu, N., Liu, J.-C., Tang, G. & Geng, W.-T. VASPKIT: A user-friendly interface facilitating high-throughput computing and analysis using VASP code. *Comput. Phys. Commun.* **267**, 108033 (2021).

11	Grimme, S., Ehrlich, S. & Goerigk, L. Effect of the damping function in dispersion corrected density functional theory. *J. Comput. Chem.* **32**, 1456-1465 (2011).

12	Kresse, G. & Furthmüller, J. Efficient iterative schemes for ab initio total-energy calculations using a plane-wave basis set. *Phys. Rev. B* **54**, 11169-11186 (1996).

13	Kresse, G. & Furthmüller, J. Efficiency of ab-initio total energy calculations for metals and semiconductors using a plane-wave basis set. *Comput. Mater. Sci.* **6**, 15-50 (1996).

14	Jain, A. *et al.* Commentary: The Materials Project: A materials genome approach to accelerating materials innovation. *APL Mater.* **1,** 011002 (2013).

15	Yin, Z. *et al.* Hybrid Catalyst Coupling Single-Atom Ni and Nanoscale Cu for Efficient CO_2_ Electroreduction to Ethylene. *J. Am. Chem. Soc.* **144**, 20931-20938 (2022).

16	He, Q. *et al.* Highly Selective CO_2_ Electroreduction to C_2_H_4_ Using a Dual-Sites Cu(II) Porphyrin Framework Coupled with Cu_2_O Nanoparticles via a Synergetic-Tandem Strategy. *Angew. Chem. Int. Ed.* **63**, e202407090 (2024).

17	Wang, M., Loiudice, A., Okatenko, V., Sharp, I. D. & Buonsanti, R. The spatial distribution of cobalt phthalocyanine and copper nanocubes controls the selectivity towards C_2_ products in tandem electrocatalytic CO_2_ reduction. *Chem. Sci.* **14**, 1097-1104 (2023).

18	Wang, L. *et al.* Integration of ultrafine CuO nanoparticles with two-dimensional MOFs for enhanced electrochemical CO_2_ reduction to ethylene. *Chin. J. Catal.* **43**, 1049-1057 (2022).

19	Zhao, Z.-H. *et al.* A Cu (111)@ metal–organic framework as a tandem catalyst for highly selective CO_2_ electroreduction to C_2_H_4_. *Chem. Commun.* **57**, 12764-12767 (2021).

20	Yan, T., Wang, P. & Sun, W. Y. Single‐Site Metal–Organic Framework and Copper Foil Tandem Catalyst for Highly Selective CO_2_ Electroreduction to C_2_H_4_. *Small* **19**, 2206070 (2023).

21	Yan, T., Wang, P., Xu, Z.-H. & Sun, W.-Y. Copper (II) frameworks with varied active site distribution for modulating selectivity of carbon dioxide electroreduction. *ACS Appl. Mater. Interfaces* **14**, 13645-13652 (2022).

22	Meng, D. L. *et al.* Highly selective tandem electroreduction of CO_2_ to ethylene over atomically isolated nickel–nitrogen site/copper nanoparticle catalysts. *Angew. Chem.* **133**, 25689-25696 (2021).

23	Wang, M. *et al.* Tandem electrocatalytic CO_2_ reduction with Fe-porphyrins and Cu nanocubes enhances ethylene production. *Chem. Sci.* **13**, 12673-12680 (2022).

24	Meng, D.-L. *et al.* Highly Selective Tandem Electroreduction of CO_2_ to Ethylene over Atomically Isolated Nickel–Nitrogen Site/Copper Nanoparticle Catalysts. *Angew. Chem. Int. Ed.* **60**, 25485-25492 (2021).

25	Shan, W. *et al.* In Situ Surface-Enhanced Raman Spectroscopic Evidence on the Origin of Selectivity in CO_2_ Electrocatalytic Reduction. *ACS Nano* **14**, 11363-11372 (2020).

26	Chernyshova, I. V., Somasundaran, P. & Ponnurangam, S. On the origin of the elusive first intermediate of CO_2_ electroreduction. *Proc. Natl. Acad. Sci.* **115**, E9261-E9270 (2018).

27	Yang, S. *et al.* In Situ Structure Refactoring of Bismuth Nanoflowers for Highly Selective Electrochemical Reduction of CO_2_ to Formate. *Adv. Funct. Mater.* **33**, 2301984 (2023).

28	Zhao, Y. *et al.* Elucidating electrochemical CO_2_ reduction reaction processes on Cu(hkl) single-crystal surfaces by in situ Raman spectroscopy. *Energy Environ. Sci.* **15**, 3968-3977 (2022).

29	Hao, J. *et al.* Strain Relaxation in Metal Alloy Catalysts Steers the Product Selectivity of Electrocatalytic CO_2_ Reduction. *ACS Nano* **16**, 3251-3263 (2022).

30	Shao, F. *et al.* In situ spectroelectrochemical probing of CO redox landscape on copper single-crystal surfaces. *Proc. Natl. Acad. Sci.* **119**, e2118166119 (2022).

31	Zhan, C. *et al.* Key intermediates and Cu active sites for CO_2_ electroreduction to ethylene and ethanol. *Nat. Energy* **9**, 1485-1496 (2024).

32	Yao, K. *et al.* Mechanistic Insights into OC–COH Coupling in CO_2_ Electroreduction on Fragmented Copper. *J. Am. Chem. Soc.* **144**, 14005-14011 (2022).
